# Nutritional guide to feeding wheat and wheat co-products to swine: a review

**DOI:** 10.1093/tas/txae106

**Published:** 2024-07-16

**Authors:** Ethan B Stas, Joel M DeRouchey, Robert D Goodband, Mike D Tokach, Jason C Woodworth, Jordan T Gebhardt

**Affiliations:** Department of Animal Sciences and Industry, Kansas State University, Manhattan, KS 66506-0201, USA; Department of Animal Sciences and Industry, Kansas State University, Manhattan, KS 66506-0201, USA; Department of Animal Sciences and Industry, Kansas State University, Manhattan, KS 66506-0201, USA; Department of Animal Sciences and Industry, Kansas State University, Manhattan, KS 66506-0201, USA; Department of Animal Sciences and Industry, Kansas State University, Manhattan, KS 66506-0201, USA; Department of Diagnostic Medicine/Pathobiology, College of Veterinary Medicine, Kansas State University, Manhattan, KS 66506-0201, USA

**Keywords:** off-quality wheat, swine, wheat, wheat by-products, wheat co-products

## Abstract

Inclusion of wheat grain can offer feeding opportunities in swine diets because of its high starch, crude protein (CP), amino acid (AA), and phosphorus (P) content. High concentrations of starch within wheat grain makes it a good energy source for swine. Mean energy content of wheat was 4,900 and 3,785 kcal/kg dry matter (DM) for digestible energy and metabolizable energy, respectively. CP concentration can vary based on the class of wheat which include hard red winter, hard red spring, soft red winter, hard white, soft white, and durum. The average CP of all wheat data collected in this review was 12.6% with a range of 8.5% to 17.6%. The AA concentration of wheat increases with increasing CP with the mean Lys content of 0.38% with a standardized ileal digestibility (SID) of 76.8%. As CP of wheat increases, the SID of AA in wheat also increases. Mean P of wheat was 0.27% and median P was 0.30%. Off-quality wheat is often associated with sprouts, low-test weight, or mycotoxin-contamination. Sprouted and low-test weight wheat are physical abnormalities associated with decreased starch within wheat kernel that leads to reductions in energy. The assumed energy value of wheat grain may need to be reduced by up to 10% when the proportion of sprouted to non-sprouted wheat is up to 40% whereas above 40%, wheat’s energy may need to be reduced by 15% to 20%. Low-test weight wheat appears to not influence pig performance unless it falls below 644 kg/m^3^ and then energy value should be decreased by 5% compared to normal wheat. Deoxynivalenol (DON) contamination is most common with wheat grain. When content is above the guidance level of 1 mg/kg of DON in the complete diet, each 1 mg/kg increase in a DON-contaminated wheat-based diet will result in a 11% and 6% reduction in ADG and ADFI for nursery pigs, and a 2.7% and 2.6% reduction in ADG and ADFI, in finishing pigs, respectively. Wheat co-products are produced from the flour milling industry. Wheat co-products include wheat bran middlings, millrun, shorts, and red dog. Wheat co-products can be used in swine diets, but application may change because of differences in the final diet energy concentration due to changes in the starch and fiber levels of each wheat co-product. However, feeding wheat co-products are being evaluated to improve digestive health. Overall, wheat and wheat co-products can be fed in all stages of production if energy and other nutrient characteristics are considered.

## Teaser

Inclusion of wheat grain and wheat co-products can provide opportunities in swine diets because of their nutritional components.

## Introduction

Because of their starch content (45% to 65%), cereals grains serve as the main energy source in swine diets. Wheat is a major cereal grain that is produced throughout the world for both human and livestock consumption. Worldwide wheat production has continued to steadily increase and totaled approximately 781 million tonnes in 2023, which ranks second among cereal grains (USDA, 2023). Wheat can offer many useful properties in swine diets because of its nutrient profile, digestibility, enzymatic activity, and pelleting characteristics (Stein et al., 2010). However, depending on the class of wheat, the nutrient content may vary.

The quality of wheat can be affected due to adverse weather conditions, delayed harvest, or improper storage resulting in “off-quality” wheat. The 3 most common occurrences are sprouted, low-test weight, and mycotoxin-contaminated wheat. Sprouted and low-test weight wheat are physical abnormalities associated with reduced energy content and possibly other nutrient alterations if severely damaged (Murray and Huber, 1968; Hines and Pollman 1982). Mycotoxins are produced by molds growing on wheat grain which can lead to reduced performance in pigs depending on the type of mycotoxin and the concentration. Although feeding off-quality wheat may result in diminished performance, it may be priced at a discount which presents an economic opportunity in swine diets (Marston and DeRouchey, 2004). However, it is important to understand handling and feeding strategies of off-quality wheat.

Wheat that is processed in flour milling intended for the human food industry produces various co-products. Wheat co-products include wheat bran, middlings, millrun, shorts, and red dog (AAFCO, 2023). Depending on the area of the world, availability of wheat co-products is variable. Wheat middlings are most common in the United States, wheat millrun is common in Canada, and wheat bran is more common in Europe. Wheat co-product classification is dependent on the combination and concentrations of different fractions within the co-products (Huang et al., 2012). Therefore, wheat co-products vary in their nutrient profile, amino acid (AA) profile, and digestibility. Clear characterization of these co-products is warranted for consistency across the industry and application into swine diets.

The first objective of this review was to provide an understanding of nutritional properties associated with wheat grain and where there may be advantages as an energy source in swine diets. The second objective of this review was to provide an understanding of the effects of feeding off-quality wheat on pig performance and how it can be effectively incorporated into swine diets. The last objective of this review was to clearly characterize wheat co-products and provide insight into potential applications in swine diets to reduce cost.

## Procedures

The current review utilized a literature search to compile previously published peer-reviewed journal articles to summarize nutrient, digestibility, and processing characteristics of wheat and wheat co-products. The search was performed via the Kansas State University Libraries under the CAB Direct and PubMed database, in addition to Google Scholar. Search terms including (WHEAT OR WHEAT CO-PRODUCTS OR WHEAT BY-PRODUCTS OR WHEAT BRAN OR WHEAT MIDDLINGS OR WHEAT MILLRUN OR WHEAT SHORTS OR WHEAT RED DOG) AND (PIGS OR SWINE) AND the respective search item of interest (cereal grain, protein, AA, phosphorus, energy, standardized ileal digestibility (SID), growth performance, nursery, finishing, sows, carcass, particle size, pelleting, pellet durability, pelleting aid, pellet quality, off-quality, sprouted, test weight, mycotoxin, deoxynivalenol, zearalenone, ochratoxin, ergot alkaloids, fumonisin, aflatoxin, fiber, starch, gastrointestinal, microbiome, and intestinal). Articles were not restricted based on region but were restricted to primarily English language. Once relative articles were identified, they were filed and categorized according to topic for further evaluation.

Wheat grain nutrient data was collected from 38 published peer-reviewed journal articles totaling 55 wheat samples. Wheat standardized ileal digestible (SID) data was obtained from 11 published peer-reviewed journal articles totaling 30 wheat samples. Wheat energy values were collected from 10 published peer-reviewed journal articles totaling 28 wheat samples. The time frame for data selection was restricted from 2013 to 2024 to provide an accurate representation of wheat based on recent literature. Additionally, there are previous publications summarizing wheat nutrient characteristics before 2013 (NRC, 2012; Rosenfelder et al., 2013). For off-quality wheat, no year restriction was enforced because of little available research. A total of 11 published peer-reviewed journal articles were collected with a total of 22 deoxynivalenol (DON)-contaminated wheat-based diets for a regression analysis on nursery and finishing pig growth performance.

Wheat co-product nutrient data was collected from 42 published peer-reviewed journal articles totaling 121 wheat co-product samples. Mean nutrient values were summarized from 28 wheat bran, 48 wheat middlings, 5 wheat millrun, 21 wheat shorts, and 19 wheat red dog samples. Data on SID of crude protein (CP) and AAs of wheat co-products was collected from 17 published peer-reviewed journal articles consisting of 5 wheat bran, 27 wheat middlings, 7 wheat shorts, and 6 wheat red dog samples. Using data from 10 journal articles, mean energy values were developed from 12 wheat bran, 21 wheat middlings, 9 wheat shorts, and 10 wheat red dog samples. The time frame for data selection was restricted from 2008 to 2024 to provide an accurate representation of wheat based on recent literature. In contrast to the wheat section, the wheat co-product literature search was extended to the last 15 years because of a relevant journal article from Nortey et al. (2008) comparing various wheat co-products.

A main objective of this review was to characterize co-product nutrient differences between wheat bran, wheat middlings, wheat millrun, wheat shorts, and wheat red dog. As publications were reviewed, the wheat co-products were initially classified based on the co-product terminology utilized in the journal article. The classification was either confirmed or re-classified based on Association of American Feed Control Officials (AAFCO; 2023) definitions and nutrient outliers. A total of 11 wheat co-product samples had to be re-classified. The basis of the reclassification to a different co-product class was due to the reported values of CF, starch, or insoluble fiber.

## Feeding Wheat Grain to Swine

### Classes of Wheat

Discussion on classes of wheat is primarily based on U.S. classification, although many other countries and regions utilize the same or similar classifications for wheat. Wheat is classified based on hardness (hard or soft), color (red or white), and growing season (winter or spring; Table 1). Hardness is an indication of protein concentration within the endosperm of the wheat kernel, specifically the protein-to-starch ratio and the physical force it takes to fracture the kernel (Saleem et al., 2022). Assuming 88% dry matter (DM), hard wheat is typically classified as having greater than 10.5% protein and soft wheat is generally lower according to U.S. Wheat Associates (2022). However, depending on various factors such as growing region, weather, cultivar, soil nitrogen, harvest time, etc, hard and soft classes of wheat can fall outside of the protein classification. The ideal protein content of wheat is based on its end use for the food industry. Typically, flour intended for bread utilizes high protein wheat (hard) whereas flour for cakes or pastries preferentially uses lower protein (soft) wheat. Wheat is also classified based on kernel color. Red wheat has a reddish hue, whereas white wheat has a sandy beige color. At harvest, red wheat is typically more resistant to sprouting in wet regions, whereas white wheat is better suited for dry conditions (Grafenauer et al., 2020). Lastly, wheat varieties can be planted and harvested at different times of the year. As the name implies, spring wheat is usually planted in the spring and harvested in the fall, whereas winter wheat is planted in the fall, becomes dormant during the winter, and is harvested the following summer (Hall and Nleya, 2019). Winter wheat requires a period of vernalization to initiate the reproductive stage following initial growth (Shourbalal et al., 2019). Winter wheat requires temperature to be less than 8 °C for vernalization in the winter followed by flowering in the spring (Amasino, 2004). The Canadian Grain Commission (2023) further classifies wheat based on growing location within the country. Canadian wheat can either be classified as eastern, northern, prairie, or western. Historically, hard red winter and soft red winter are most common for livestock feed, while hard red spring may also be fed depending on the year (USDA, 2023). Western red spring wheat is the most common wheat fed in Canada and represents approximately 62% of total wheat production (Agriculture and Agri-Food Canada, 2024).

**Table 1.** United States wheat classification^1^

	Class					
	Hard red winter	Hard red spring	Soft red winter	Hard white	Soft white	Durum
Protein range (%) 12% moisture basis	10.0 to 13.0	12.0 to 15.0	8.5 to 10.5	10.0 to 14.0	8.5 to 10.5	12.0 to 15.0
Growing region (top 5 producing)	Kansas Oklahoma Montana South Dakota Texas	North Dakota Minnesota Montana Idaho South Dakota	Illinois Ohio Kentucky Tennessee Missouri	Kansas Nebraska Colorado Utah California	Washington Idaho Oregon Michigan Utah	North Dakota Montana Arizona California Idaho
Planting season	Fall	Spring	Fall	Fall or spring	Fall or spring	Spring
Harvest season	Summer	Fall	Summer	Summer or fall	Summer or fall	Fall
Application	Asian noodles Hard rolls Flat breads Flour Cereal	Hearth breads Rolls Croissants Bagels Pizza crust	Cookies Crackers Pretzels Pastries Flat bread	Asian noodles Pan breads Flat breads Whole wheat	Cakes Patries Asian noodles Flat breads	Pasta Breads
Production (million metric tons)	15.93	11.23	11.98	0.51	5.98	1.56
Production (% of all wheat)	33.8	23.8	25.4	1.1	12.7	3.3
Subclasses	None	Dark northern spring Northern spring Red spring	None	None	Soft white White club Western white	Hard amber durum Amber durum Durum

^1^ USDA, 2023 and U.S. Wheat Associates, 2023.

**Figure 1.**
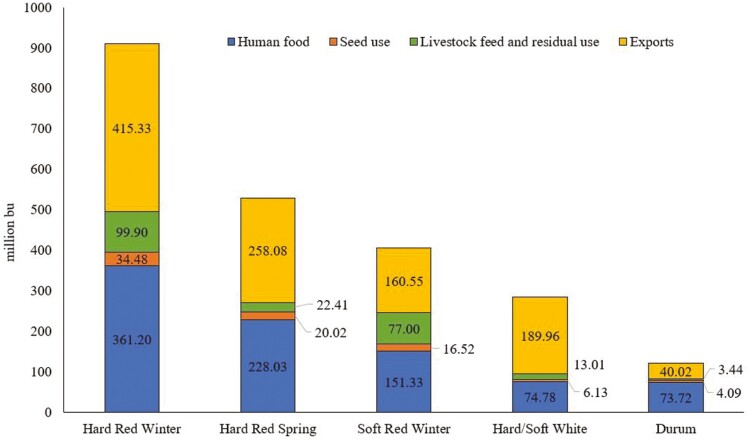
United States wheat class production by end use (USDA, 2023).

**Figure 2.**
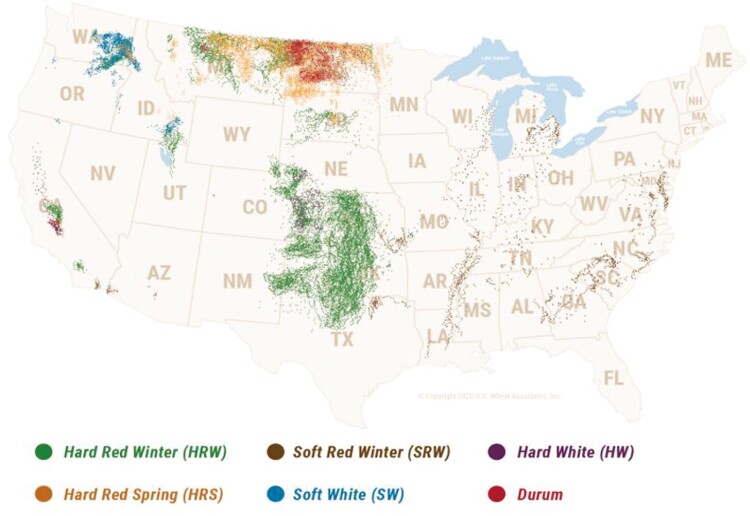
United States wheat class by state (U.S. Wheat Associates, 2022. The map is a representation of wheat class production density by geographical location).

**Figure 3.**
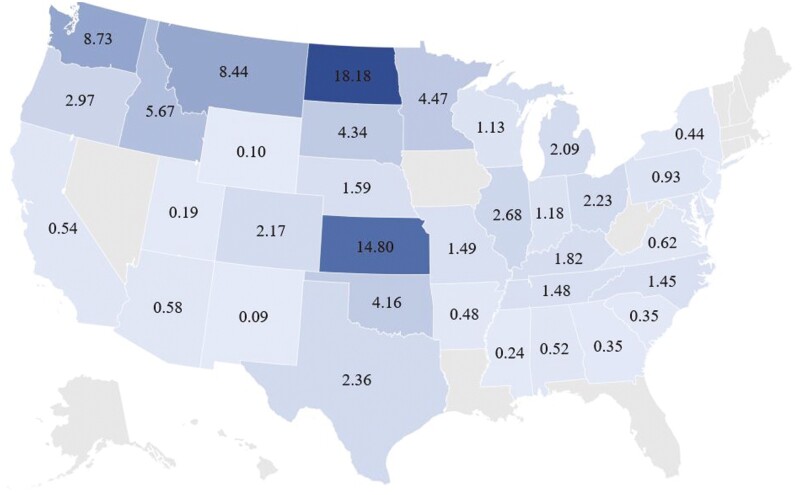
Percentage of United States wheat production by state (USDA, 2023).

Hard red winter wheat is the most common wheat class in the US representing approximately 34% of all wheat production (Figure 1; USDA, 2023). It is grown in a variety of areas across the country including the great plains (Kansas, Oklahoma, Texas, and North Dakota), the pacific northwest (Montana and Washington), and California (Figures 2 and 3). It is utilized either alone or in combination with other classes of wheat for general all-purpose flour. Hard red winter wheat provides consistency for the milling process because of its availability and nutritional properties (U.S. Wheat Associates, 2022).

Hard red spring wheat represents approximately 24% of all U.S. wheat production (USDA, 2023) which is mostly grown in the north central region of the US (Montana and North Dakota). Hard red spring is known for its high protein content ranging from 12% to 15% protein making it an appealing wheat class for hard breads such as bagels and pizza crust. It also provides improved flour yield relative to other wheat classes because of its dense endosperm. Hard red spring wheat is further divided into subclasses consisting of dark northern spring, northern spring, and red spring. These subclasses are an indication of the percentage of dark, hard, vitreous kernels present (U.S. Wheat Associates, 2022).

For the eastern third of the US, soft red winter wheat is the most common class of wheat representing 25% of all wheat production (USDA, 2023). This class of wheat is primarily grown in Illinois, Indiana, Arkansas, North Carolina, and many other eastern states. The soft endosperm of soft red winter wheat results in a lower protein content of 8.5% to 10.5%. However, soft red winter wheat is often blended with hard red classes of wheat to improve the texture and appearance of the products it is used to produce (U.S. Wheat Associates, 2022).

Soft white wheat represents approximately 13% of all wheat production (USDA, 2023). It is mostly found in the pacific northwest (Washington, Idaho, and Oregon) and consists of 3 subclasses. Subclasses are an indication of the amount of club wheat present and include soft white, white club, and western white. Club wheat is a subspecies that is blended with conventional soft white wheat to form subclasses (Johnson et al., 2008). An appealing quality of soft white wheat is a low moisture content which is typically less than 10%. Its high DM allows for optimization of flour extraction, reduced particle size, and appearance of its end products (U.S. Wheat Associates, 2022). However, similar to soft red winter wheat, protein is lower and ranges from 8.5% to 10.5%.

The least common class of wheat in the US is hard white wheat only representing 1% of all wheat production (USDA, 2023). Hard white wheat has both winter and spring varieties which are mostly grown in western Kansas and Nebraska. Hard white wheat can have a high protein content containing up to 14% depending on the growing season. Hard white wheat is associated with decreased polyphenol oxidase compared to other wheat classes (Park et al., 1997). Polyphenol oxidase is an enzyme that is responsible for undesirable browning of wheat products (Demeke and Morris, 2002).

The last class of wheat is durum, the hardest of all wheat varieties. Durum’s protein ranges from 12% to 15% making it an appealing wheat variety for human consumption. It is an excellent class for pasta production. Durum production represents only 3.3% of all U.S. wheat production (USDA, 2023). Durum is mostly grown in North Dakota, but some varieties can also be found in Arizona and California often referred to as desert durum. Subclasses are an indication of the percentage of hard, vitreous, amber colored kernels which include hard amber durum, amber durum, or durum if it fails to meet the other 2 subclass criteria (U.S. Wheat Associates, 2023).

### Standards for Wheat

Wheat is graded on a scale of 1 to 5 with 1 representing high quality wheat and 5 representing poor quality wheat (Table 2; USDA, 2014). Factors affecting the U.S. grade of wheat in a single lot include the class of wheat, contrasting classes of wheat, damaged kernels, dockage, foreign material, other grains, and sieve size. The minimum test weight affects the U.S. grade depending on the class of wheat. For example, hard red winter must meet a test weight of 60.0 lb/bu (772 kg/m^3^) to qualify for U.S. grade of 1, whereas hard red spring wheat only requires a test weight of 58.0 lb/bu (747 kg/m^3^) for the same grade. A greater percentage of a contrasting class of wheat present will also result in a poorer U.S. grade. For example, hard red winter wheat and soft white wheat are contrasting classes. Therefore, if there is 2% hard red winter wheat within a soft white wheat purchase, the U.S. grade will depreciate from 1 to 2. Similarly, presence of damaged kernels, which can include defects, heat damage, shrunken, and broken kernels, will negatively impact the U.S. grade. Foreign material present will also result in a poorer grade. Foreign material is anything other than the wheat kernels including the presence of other grains. Rescreening or recleaning wheat to remove substances other than wheat can be done to improve the U.S. grade. In addition, near-infrared spectroscopy (NIRS) has emerged as an affordable and reliable method to sort wheat grain by grade (Johnson, 2020). The final factor that affects the U.S. grade is its ability to fit through a 0.064 × 0.375-inch (0.163 × 0.953 cm) oblong-hole sieve. Ultimately, the grade wheat receives will influence its end use whether it is exported, enters the milling process, or is used for livestock.

**Table 2.** USDA grades and grade requirements for all classes and subclasses of wheat^1^

Grading factors	Grade U.S. Nos.				
	1	2	3	4	5
Minimum limits (lb/bu)					
Test weight					
Hard red spring wheat or white club wheat	58.0	57.0	55.0	53.0	50.0
All other classes and subclasses	60.0	58.0	56.0	54.0	51.0
Maximum limits (%)					
Damaged kernels					
Heat (part of total)	0.2	0.2	0.5	1.0	3.0
Total	2.0	4.0	7.0	10.1	15.0
Foreign material	0.4	0.7	1.3	3.0	5.0
Shrunken and broken kernels	3.0	5.0	8.0	12.0	20.0
Total^2^	3.0	5.0	8.0	12.0	20.0
Wheat of other classes	10.0	10.0	10.0	10.0	10.0
Contrasting classes	1.0	2.0	3.0	10.0	10.0
Total^3^	3.0	5.0	10.0	10.0	10.0
Stones	0.1	0.1	0.1	0.1	0.1
Maximum count limits^4^					
Animal filth	1	1	1	1	1
Castor beans	1	1	1	1	1
Crotalaria seeds	2	2	2	2	2
Glass	0	0	0	0	0
Stones	3	3	3	3	3
Unknown foreign substances	3	3	3	3	3
Total^5^	4	4	4	4	4
Insect-damaged kernels in 100 g	31	31	31	31	31

^1^ USDA, 2014. Does not include mixed wheat.

^2^Includes damaged kernels, foreign material, shrunken, and broken kernels.

^3^Includes contrasting classes.

^4^Any wheat purchase that exceeds the maximum count limit is not eligible for a U.S. grade.

^5^Includes any combination of animal filth, castor beans, crotalaria seeds, glass, stones, or unknown foreign substance.

### Nutrient Profile

#### Energy

Wheat is considered to be a high starch, low fiber cereal grain making it a good energy source for swine diets (McGhee and Stein, 2020). Digestible carbohydrates include monosaccharides, disaccharides, and polysaccharides such as starch. Starch in wheat is comprised of approximately 20% amylose and 80% amylopectin (Kiem et al., 2014). Non-digestible carbohydrates include oligosaccharides, resistant starch, and non-starch polysaccharides (fiber). On average, wheat is approximately 10.9% total dietary fiber (TDF), most of which is insoluble dietary fiber. Wheat does contain β-glucans and arabinoxylans, which are soluble fibers that have been shown to have beneficial effects on acetate and lactate production within digestive tract of swine (Cervantes-Pahm et al., 2014a; Bai et al., 2021).

Mean gross energy (GE), digestible energy (DE), and metabolizable energy (ME) values of wheat were 4,419, 3,393, and 3,831 kcal/kg of DM, respectively. The DE of wheat ranged from 3,456 to 4,126 kcal/kg DM and ME ranged from 3,353 to 3,975 kcal/kg DM. Previous literature suggested wheat contains 91% to 97% of the energy of corn for pigs (Stein et al., 2010). However, results based on recent data suggest wheat’s energy is approximately 99% and 98% of the energy of corn for DE and ME, respectively. Similar to protein, energy can be influenced by many factors associated with wheat. Rosenfelder et al. (2013) summarized 15 studies and observed that winter wheat had greater DE, but lower ME compared to spring wheat. The author’s suggested these differences may have been associated with increased variation due to methodology, wheat class, and growing conditions. In the current review, there appears to be no correlation between protein (hardness) and DE or ME in wheat. A regression analysis revealed CP was not significantly related (*P *> 0.10) to the DE or ME of the 28 samples of wheat collected from the 10 published peer-reviewed journal articles.

There were no publications found that determined net energy (NE) from the current review’s search criteria and restrictions imposed. However, NE can still be calculated from publications that report DE, EE, starch, CP, and ADF values by utilizing equations 1 to 8 from NRC (2012). The average NE from 16 wheat samples across 5 journal publications was calculated to be 2,786 kcal/kg DM which agrees with previously published wheat NE values. The NRC (2012) reports an NE of 2,788 and 3,004 kcal/kg DM for hard red and soft red wheat, respectively. The CVB (2016) reports wheat NE at 2,888 kcal/kg DM and the INRA (2021) reports NE at 2,900 kcal/kg DM for soft wheat.

### CP and AAs

The CP of wheat samples collected from literature ranged from 8.5% to 17.6% on an as-fed basis (Table 3). Wheat classifications varied among publications with the majority not indicating the wheat class, origin, or region utilized for the study. It is often difficult to classify wheat as hard or soft based on only CP. U.S. Wheat Associates (2022) classifies soft and hard wheat as below or above 10.5% CP, respectively, whereas the NRC (2012) utilizes 11% CP as the separation of classification. In addition, often there is overlap with CP content and wheat classes. For example, Rosenfelder et al. (2015) reported that 7 out of 8 soft wheat samples had greater than 11% CP. In addition, Bloxham et al. (2018) utilized hard red wheat, but one sample only contained 10.2% CP. Factors such as weather, geographic location, and growing season can also influence protein content. Winter wheat is associated with higher yields than spring wheat, which is negatively correlated with protein concentration (Posner, 2000). Therefore, spring wheat classes will generally have higher protein than winter wheat classes. However, varying climate conditions can influence these observations. Many factors can influence the nutrient concentrations of wheat; therefore, nutrient analysis should be performed regularly.

**Table 3.** Chemical composition and SID of nutrients, AAs, and energy content in wheat (as-fed basis)^1^

Item		Chemical composition (%)			SID (%)			
	N^2^	Mean ± SD	Min	Max	N^2^	Mean ± SD	Min	Max
Nutrient composition, %								
DM	36	88.9 ± 1.62	86.5	92.9				
CP	55	12.6 ± 1.75	8.5	17.6	28	87.2 ± 4.75	75.0	90.4
EE	52	1.7 ± 0.55	0.7	3.8				
Ash	49	1.6 ± 0.24	0.7	2.1				
ADF	49	2.8 ± 0.77	0.8	5.8				
NDF	49	11.7 ± 2.79	8.1	23.1				
TDF	16	10.9 ± 1.68	9.2	15.2				
Ca	30	0.07 ± 0.046	0.02	0.19				
P	30	0.27 ± 0.104	0.07	0.45				
Starch	39	57.7 ± 5.99	30.3	64.4				
Indispensable AA, %								
Arg	43	0.56 ± 0.055	0.47	0.71	30	88.4 ± 4.51	80.6	99.8
His	43	0.34 ± 0.082	0.22	0.53	30	88.2 ± 4.51	80.9	94.1
Ile	45	0.43 ± 0.066	0.30	0.57	30	87.1 ± 4.25	78.4	94.0
Leu	43	0.84 ± 0.141	0.53	1.18	29	88.1 ± 3.91	80.5	94.8
Lys	45	0.38 ± 0.059	0.30	0.53	30	76.8 ± 7.95	63.7	89.9
Met	45	0.19 ± 0.028	0.14	0.26	30	88.3 ± 3.39	82.9	94.5
Phe	43	0.58 ± 0.108	0.42	0.84	30	89.2 ± 4.20	82.3	96.0
Thr	45	0.35 ± 0.041	0.27	0.44	30	80.9 ± 5.80	65.4	90.8
Trp	43	0.15 ± 0.028	0.08	0.21	28	86.5 ± 4.92	76.8	94.6
Val	45	0.56 ± 0.113	0.39	0.83	30	85.0 ± 4.41	75.1	92.9
Dispensable AA, %								
Ala	39	0.47 ± 0.079	0.37	0.64	29	80.0 ± 7.04	61.7	90.8
Asp	39	0.63 ± 0.071	0.52	0.79	29	80.7 ± 6.65	66.8	91.5
Cys	39	0.31 ± 0.065	0.20	0.46	29	89.2 ± 3.85	78.3	95.1
Glu	39	3.45 ± 0.582	2.54	5.16	29	94.8 ± 2.15	88.9	97.5
Gly	38	0.50 ± 0.050	0.41	0.64	28	85.3 ± 8.08	63.8	96.4
Pro	38	1.28 ± 0.254	0.84	1.93	27	109.9 ± 45.47	71.7	328.0
Ser	39	0.53 ± 0.083	0.25	0.71	29	89.3 ± 3.59	80.3	94.7
Tyr	28	0.16 ± 0.125	0.02	0.41	17	90.9 ± 6.51	77.1	99.5
Energy content, kcal/kg DM								
GE	45	4,401 ± 100.2	3,954	4,604				
DE	27	3,900 ± 144.5	3,456	4,126				
ME	28	3,785 ± 132.8	3,353	3,975				
Calculated NE^3^	15	2,786 ± 60.0	2,672	2,860				

^1^Literature cited: Abelilla and Stein, 2019; Bloxham et al., 2018; Bolarinwa and Adeola, 2016; Cervantes-Pahm et al., 2014a, 2014b; De Jong et al., 2016; Eklund et al., 2014; Fan et al., 2017; Heyer et al., 2022; Jaworski et al., 2015; Jeon et al., 2019; Koo et al., 2018; Lee et al., 2020; Le et al., 2016; McGhee and Stein, 2018; McGhee and Stein, 2020; Oliveria et al., 2020; Pan and An, 2020; Park et al., 2016; Rodriguez et al., 2020; Rosenfelder et al., 2015; Schemmer et al., 2020; She et al., 2015; Tan et al., 2021; Wang et al., 2018; Xie et al., 2017; Zhang et al., 2020; Zhao et al., 2019; Zhou et al., 2016.

^2^Number of samples of wheat from collected publications utilized for mean values.

^3^Calculated using equations 1 to 8 from NRC (2012). Net energy (kcal/kg DM) = (0.700 × DE) + (1.61 × EE) + (0.48 × Starch) − (0.91 × CP)− (0.87 × ADF).

**Figure 4.**
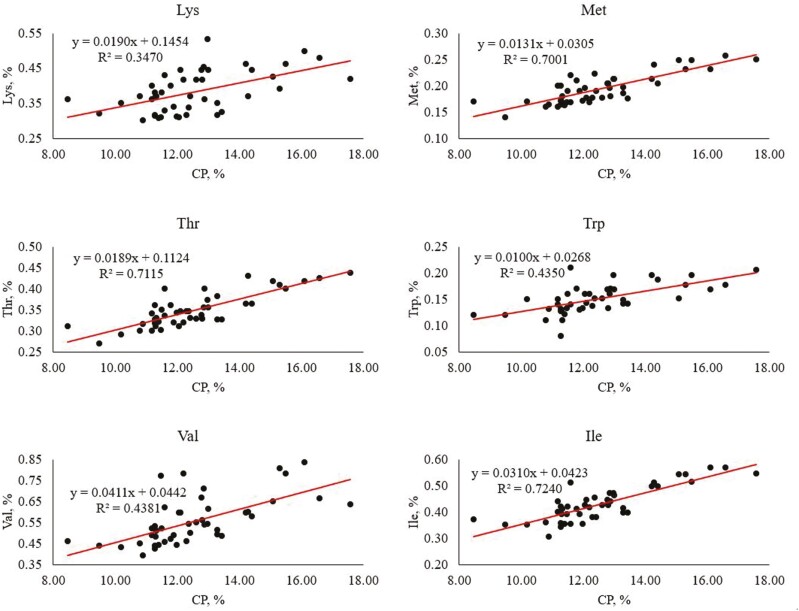
Simple linear regression to predict total Lys, Met, Thr, Trp, Val, and Ile concentration using the CP content in wheat (literature cited: Abelilla and Stein, 2019; Bloxham et al., 2018; Bolarinwa and Adeola, 2016; Cervantes-Pahm et al., 2014b; De Jong et al., 2014; Eklund et al., 2014; Fan et al., 2017; Heyer et al., 2022; Jaworski et al., 2015; Jeon et al., 2019; Koo et al., 2018; Lee et al., 2020; Le et al., 2016; McGhee and Stein, 2018; McGhee and Stein, 2020; Oliveria et al., 2020; Pan and An, 2020; Park et al., 2016; Rodriguez et al., 2020; Rosenfelder et al., 2015; Schemmer et al., 2020; She et al., 2015; Tan et al., 2021; Wang et al., 2018; Xie et al., 2017; Zhang et al., 2020; Zhao et al., 2019; Zhou et al., 2016. There were 45 samples for all AA except Trp which had 43 samples).

The average CP content across all summarized wheat data was 12.6%. As CP increased within wheat samples, so did AA content. Therefore, a simple linear regression model was developed to estimate the AA concentration of a sample of wheat based on CP (Table 4). All AA regression coefficients were significant (*P *< 0.001) except for Tyr. All coefficients for the explanatory variable (CP) were positive, indicating an increase in CP will result in an increase in each AA. The coefficient for CP on Lys is 0.019, meaning a 1% increase in CP will result in a 0.019% increase in Lys (Figure 4). Indicated by the *R*^2^ value, 33.1% of the variation in Lys concentration is explained by wheat’s CP content. This is also the lowest coefficient of determination of all AA. For other indispensable AA, a change in CP will have the greatest unit change in Leu (0.051) and the least change in Trp (0.010). For dispensable AA, a change in CP of wheat will result in the greatest unit change in Glu (0.306) and the least change in Cys (0.026). The variation that is most explained by CP was Ile (72.4%) and Glu (75.9%) for indispensable and dispensable AA, respectively. The variation that is least explained by CP was Cys (43.2%) for dispensable AA. These models can be utilized to predict the approximate AA profile of wheat based on percent CP. However, the models will only provide an estimate of AA content based on CP and should be used with caution.

**Table 4.** Simple linear regression to predict AAs concentration using the CP content in wheat^1^

Item	*N* ^2^	Intercept	Slope (CP)	*R* ^2^	Standard error	*P*
Indispensable AA						
Arg	43	0.273	0.023	0.598	0.036	<0.001
His	43	−0.059	0.032	0.506	0.058	<0.001
Ile	45	0.042	0.031	0.724	0.035	<0.001
Leu	43	0.207	0.051	0.439	0.107	<0.001
Lys	45	0.145	0.019	0.347	0.048	<0.001
Met	45	0.031	0.013	0.700	0.016	<0.001
Phe	43	−0.037	0.049	0.697	0.060	<0.001
Thr	45	0.112	0.019	0.711	0.022	<0.001
Trp	43	0.027	0.010	0.435	0.021	<0.001
Val	45	0.044	0.041	0.438	0.085	<0.001
Dispensable AA						
Ala	39	0.048	0.034	0.522	0.055	<0.001
Asp	39	0.245	0.031	0.542	0.049	<0.001
Cys	39	−0.009	0.026	0.432	0.049	<0.001
Glu	39	−0.308	0.306	0.759	0.289	<0.001
Gly	38	0.173	0.027	0.644	0.030	<0.001
Pro	38	−0.215	0.122	0.647	0.153	<0.001
Ser	39	0.105	0.035	0.471	0.061	<0.001
Tyr	28	0.412	−0.020	0.074	0.122	0.161

^1^Literature cited: Abelilla and Stein, 2019; Bloxham et al., 2018; Bolarinwa and Adeola, 2016; Cervantes-Pahm et al., 2014b; De Jong et al., 2016; Eklund et al., 2014; Fan et al., 2017; Heyer et al., 2022; Jaworski et al., 2015; Jeon et al., 2019; Koo et al., 2018; Lee et al., 2020; Le et al., 2016; McGhee and Stein, 2018; McGhee and Stein, 2020; Oliveria et al., 2020; Pan and An, 2020; Park et al., 2016; Rodriguez et al., 2020; Rosenfelder et al., 2015; Schemmer et al., 2020; She et al., 2015; Tan et al., 2021; Wang et al., 2018; Xie et al., 2017; Zhang et al., 2020; Zhao et al., 2019; Zhou et al., 2016.

^2^Number of samples of wheat from collected publications utilized for mean values.

The mean total starch was 57.7% across all wheat samples from collected literature. As total starch of a wheat sample increased, CP decreased. Starch is a main determinant of wheat protein content. If starch granules do not fill up, structural protein content is indirectly increased (Lalman and Highfill, 2002), both having a major effect on grain energy value. Shang et al. (2020) observed that soft wheat varieties (lower CP) had a larger proportion of total starch compared to hard wheat varieties across 12 wheat samples. For other nutrients such as DM, ether extract (EE), TDF, acid-detergent fiber (ADF), and neutral detergent fiber (NDF), mean values from the current review were similar to previously reported values (Stein et al., 2010; NRC, 2012; Rosenfelder et al., 2013).

Wheat comparisons to other common cereal grains were accomplished by utilizing published journal articles with direct comparisons of wheat to either corn (Table 5), barley (Table 6), or sorghum (Table 7). Overall, wheat is a good source of CP and AA compared to other common cereal grains.

**Table 5.** Nutrient and AA composition and SID of wheat compared to corn (as-fed basis)^1^

	Chemical composition (%, Mean ± SD)				SID (%, Mean ± SD)			
Item	*N* ^2^	Wheat	Corn	Ratio	*N* ^2^	Wheat	Corn	Ratio
Nutrient composition, %								
DM	16	89.1 ± 1.62	88.0 ± 1.22	1.01				
CP	16	12.3 ± 1.85	7.9 ± 1.36	1.56	7	84.6 ± 6.92	81.8 ± 10.13	1.03
EE	15	2.1 ± 0.66	3.2 ± 0.57	0.66				
Ash	15	1.7 ± 0.22	1.3 ± 0.25	1.31				
ADF	11	3.0 ± 0.50	2.5 ± 0.73	1.20				
NDF	11	12.8 ± 3.10	10.3 ± 3.22	1.24				
Ca	6	0.11 ± 0.063	0.06 ± 0.060	1.83				
P	6	0.38 ± 0.055	0.24 ± 0.028	1.58				
Starch	9	56.7 ± 5.09	60.0 ± 4.87	0.95				
Indispensable AA, %								
Arg	10	0.56 ± 0.063	0.33 ± 0.019	1.70	8	86.1 ± 4.37	86.9 ± 9.35	0.99
His	10	0.27 ± 0.052	0.21 ± 0.020	1.29	8	85.4 ± 3.20	84.8 ± 4.66	1.01
Ile	10	0.41 ± 0.059	0.26 ± 0.031	1.58	8	82.7 ± 3.84	82.4 ± 7.12	1.00
Leu	10	0.72 ± 0.089	0.81 ± 0.108	0.89	7	84.6 ± 3.25	88.1 ± 4.57	0.96
Lys	10	0.37 ± 0.044	0.25 ± 0.029	1.48	8	72.5 ± 5.66	74.7 ± 10.59	0.97
Met	10	0.19 ± 0.027	0.15 ± 0.012	1.27	8	85.8 ± 2.30	88.4 ± 5.14	0.97
Phe	10	0.53 ± 0.096	0.39 ± 0.131	1.36	8	85.9 ± 2.98	85.9 ± 4.70	1.00
Thr	10	0.34 ± 0.041	0.26 ± 0.026	1.31	8	75.8 ± 6.21	75.8 ± 9.42	1.00
Trp	9	0.14 ± 0.024	0.06 ± 0.004	2.33	7	83.8 ± 4.52	74.3 ± 9.78	1.13
Val	10	0.51 ± 0.061	0.35 ± 0.037	1.46	8	79.9 ± 3.67	80.5 ± 6.71	0.99
Dispensable AA, %								
Ala	10	0.42 ± 0.039	0.52 ± 0.050	0.81	8	74.2 ± 6.71	84.4 ± 6.83	0.88
Asp	10	0.61 ± 0.067	0.50 ± 0.066	1.22	8	75.4 ± 5.70	80.9 ± 6.86	0.93
Cys	10	0.26 ± 0.020	0.17 ± 0.032	1.53	8	85.1 ± 3.56	80.2 ± 6.59	1.06
Glu	10	3.19 ± 0.668	1.28 ± 0.130	2.49	8	92.8 ± 1.66	87.2 ± 4.55	1.06
Gly	9	0.46 ± 0.018	0.29 ± 0.016	1.59	7	82.2 ± 12.08	80.2 ± 17.95	1.02
Pro	9	1.10 ± 0.231	0.62 ± 0.061	1.77	6	114.5 ± 22.77	99.8 ± 53.20	1.15
Ser	10	0.49 ± 0.077	0.33 ± 0.022	1.48	8	85.7 ± 2.71	83.5 ± 6.98	1.03
Tyr	7	0.29 ± 0.058	0.21 ± 0.020	1.38	4	84.6 ± 5.08	80.4 ± 4.31	1.05
Energy content (kcal/kg DM)								
GE	6	4,404 ± 174.9	4,418 ± 181.5	1.00				
DE	6	3,824 ± 218.1	3,882 ± 212.8	0.99				
ME	6	3,690 ± 206.7	3,780 ± 199.8	0.98				

^1^Mean values for each comparison are sourced from articles that have direct comparisons of wheat and corn. Literature cited: Abelilla and Stein, 2019; Cervantes-Pahm et al., 2014a, 2014b; Fan et al., 2017; Jaworski et al., 2015; Jeon et al., 2019; McGhee and Stein, 2018; McGhee and Stein 2020; Oliveria et al., 2020; Pan and An, 2020; Park et al., 2016; Rodriguez et al., 2020; She et al., 2015; Tan et al., 2021; Xie et al., 2017; Zhang et al., 2020.

^2^Number of wheat/corn comparisons from collected publications.

**Table 6.** Nutrient and AA composition and SID of wheat compared to barley (as-fed basis)^1^

	Chemical composition (%, Mean ± SD)				SID (%, Mean ± SD)			
Item	*N* ^2^	Wheat	Barley	Ratio	*N* ^2^	Wheat	Barley	Ratio
Nutrient composition, %								
DM	12	88.8 ± 1.60	88.9 ± 1.68	1.00				
CP	12	12.5 ± 2.34	11.2 ± 1.56	1.12	4	86.4 ± 7.59	80.7 ± 6.16	1.07
EE	11	1.9 ± 0.86	1.9 ± 0.56	1.00				
Ash	11	1.5 ± 0.34	1.9 ± 0.58	0.79				
ADF	11	3.1 ± 0.96	5.7 ± 2.07	0.54				
NDF	11	14.1 ± 4.06	19.0 ± 5.08	0.74				
Ca	7	0.05 ± 0.039	0.07 ± 0.033	0.71				
P	7	0.34 ± 0.038	0.33 ± 0.027	1.03				
Starch	7	58.0 ± 3.82	55.9 ± 6.34	1.04				
Indispensable AA, %								
Arg	8	0.58 ± 0.078	0.52 ± 0.067	1.12	4	92.3 ± 5.69	85.1 ± 4.40	1.08
His	8	0.29 ± 0.064	0.25 ± 0.037	1.16	4	86.5 ± 3.11	82.8 ± 2.49	1.04
Ile	8	0.44 ± 0.085	0.40 ± 0.075	1.10	4	84.3 ± 3.77	78.1 ± 4.14	1.08
Leu	8	0.77 ± 0.173	0.74 ± 0.171	1.04	3	84.8 ± 3.53	81.7 ± 3.59	1.04
Lys	8	0.39 ± 0.052	0.43 ± 0.058	0.91	4	77.6 ± 4.97	72.6 ± 6.08	1.07
Met	8	0.20 ± 0.041	0.18 ± 0.028	1.11	4	87.2 ± 2.54	80.7 ± 4.66	1.08
Phe	8	0.58 ± 0.139	0.58 ± 0.120	1.00	4	86.3 ± 2.59	82.9 ± 3.28	1.04
Thr	8	0.36 ± 0.061	0.36 ± 0.056	1.00	4	76.9 ± 7.70	75.0 ± 4.65	1.03
Trp	8	0.14 ± 0.048	0.11 ± 0.023	1.27	4	88.8 ± 3.66	83.9 ± 2.99	1.06
Val	8	0.54 ± 0.086	0.55 ± 0.092	0.98	4	80.6 ± 3.67	77.9 ± 4.27	1.03
Dispensable AA, %								
Ala	6	0.43 ± 0.056	0.42 ± 0.031	1.02	4	76.0 ± 9.90	70.9 ± 7.97	1.07
Asp	6	0.62 ± 0.087	0.61 ± 0.029	1.02	4	77.4 ± 7.22	72.9 ± 4.91	1.06
Cys	6	0.27 ± 0.051	0.23 ± 0.027	1.17	4	86.5 ± 3.23	80.4 ± 4.91	1.08
Glu	6	3.18 ± 0.812	2.35 ± 0.212	1.35	4	92.3 ± 2.48	86.7 ± 2.55	1.06
Gly	5	0.45 ± 0.033	0.34 ± 0.168	1.32	3	85.2 ± 18.58	71.5 ± 21.22	1.19
Pro	5	1.04 ± 0.269	1.01 ± 0.146	1.03	2	221.9 ± 150.12	95.0 ± 15.63	2.34
Ser	6	0.44 ± 0.117	0.37 ± 0.052	1.19	4	85.7 ± 2.71	80.6 ± 4.50	1.06
Tyr	6	0.32 ± 0.067	0.28 ± 0.058	1.14	4	84.6 ± 5.08	79.4 ± 4.20	1.07
Energy content (kcal/kg DM)								
GE	5	4,419 ± 130.8	4,439 ± 145.6	1.00				
DE	6	3,939 ± 109.9	3,699 ± 250.8	1.06				
ME	7	3,831 ± 107.6	3,577 ± 237.8	1.07				

^1^Mean values are sourced from articles that have direct comparisons of wheat and barley. Literature cited: Bolarinwa and Adeola, 2016; Cervantes-Pahm et al., 2014a, 2014b; Heyer et al., 2022; Koo et al., 2018; Lee et al., 2020; McGhee and Stein, 2018; McGhee and Stein, 2020; Park et al., 2016; Schemmer et al., 2020; Tan et al., 2021; Xie et al., 2017; Zhou et al., 2016.

^2^Number of wheat/barley comparisons from collected publications.

**Table 7.** Nutrient and AA composition and SID of wheat compared to sorghum (as-fed basis)^1^

	Chemical composition (%, Mean ± SD)				SID (%, Mean ± SD)			
Item	*N* ^2^	Wheat	Sorghum	Ratio	*N* ^2^	Wheat	Sorghum	Ratio
Nutrient composition, %								
DM	8	89.5 ± 1.75	88.7 ± 1.61	1.01				
CP	8	11.8 ± 1.17	9.3 ± 1.12	1.27	3	84.9 ± 8.89	78.5 ± 11.46	1.08
EE	8	2.1 ± 0.59	2.9 ± 0.69	0.72				
Ash	7	1.6 ± 0.29	1.3 ± 0.40	1.23				
ADF	6	2.9 ± 0.54	2.9 ± 0.50	1.00				
NDF	6	13.2 ± 4.01	9.8 ± 3.70	1.35				
Ca	2	0.12 ± 0.099	0.03 ± 0.014	4.00				
P	2	0.37 ± 0.113	0.27 ± 0.007	1.37				
Starch	6	56.7 ± 5.78	62.4 ± 6.82	0.91				
Indispensable AA, %								
Arg	6	0.54 ± 0.051	0.32 ± 0.038	1.69	3	87.3 ± 0.76	83.0 ± 8.30	1.05
His	6	0.26 ± 0.032	0.20 ± 0.044	1.30	3	85.9 ± 1.71	75.4 ± 1.67	1.14
Ile	6	0.39 ± 0.032	0.34 ± 0.051	1.15	3	83.5 ± 4.75	78.3 ± 4.18	1.07
Leu	6	0.74 ± 0.082	1.09 ± 0.160	0.68	3	85.0 ± 3.82	81.7 ± 4.72	1.04
Lys	6	0.34 ± 0.022	0.22 ± 0.067	1.55	3	75.7 ± 3.29	71.8 ± 5.06	1.05
Met	6	0.17 ± 0.019	0.14 ± 0.015	1.21	3	86.6 ± 2.24	81.6 ± 4.03	1.06
Phe	6	0.51 ± 0.073	0.43 ± 0.054	1.19	3	87.2 ± 2.86	71.3 ± 16.21	1.22
Thr	6	0.32 ± 0.043	0.27 ± 0.029	1.19	3	75.7 ± 9.60	73.2 ± 4.99	1.03
Trp	6	0.14 ± 0.021	0.08 ± 0.020	1.75	3	83.2 ± 2.16	76.1 ± 2.43	1.09
Val	6	0.48 ± 0.036	0.44 ± 0.067	1.09	3	80.1 ± 5.35	77.7 ± 3.78	1.03
Dispensable AA, %								
Ala	6	0.40 ± 0.020	0.72 ± 0.082	0.56	3	73.4 ± 10.77	79.8 ± 7.03	0.92
Asp	6	0.59 ± 0.056	0.54 ± 0.052	1.09	3	75.6 ± 7.97	77.7 ± 5.60	0.97
Cys	6	0.25 ± 0.027	0.17 ± 0.031	1.47	3	86.8 ± 3.63	73.6 ± 4.61	1.18
Glu	6	3.08 ± 0.500	1.81 ± 0.430	1.70	3	93.5 ± 1.39	82.4 ± 4.69	1.13
Gly	6	0.45 ± 0.025	0.29 ± 0.054	1.55	3	82.4 ± 16.16	74.7 ± 22.6	1.10
Pro	6	1.09 ± 0.210	0.64 ± 0.142	1.70	2	133.5 ± 5.37	137.0 ± 9.26	0.97
Ser	5	0.46 ± 0.128	0.34 ± 0.024	1.35	3	86.8 ± 3.62	81.6 ± 2.73	1.06
Tyr	4	0.31 ± 0.059	0.27 ± 0.078	1.15	1	77.1	70.8	1.09
Energy content (kcal/kg DM)								
GE	5	4,341 ± 116.5	4,368 ± 145.9	0.99				
DE	6	3,913 ± 115.7	3,959 ± 152.8	0.99				
ME	6	3,790 ± 128.5	3,851 ± 178.3	0.98				

^1^Mean values are sourced from articles that have direct comparisons of wheat and sorghum. Literature cited: Abelilla and Stein, 2019; Bolarinwa and Adeola, 2016; Cervantes-Pahm et al., 2014a, 2014b; Jaworski et al., 2015; McGhee and Stein, 2020; Pan and An, 2020; Rodriguez et al., 2020; Xie et al., 2017.

^2^Number of wheat/sorghum comparisons from collected publications.

### SID of CP and AAs

Generally, mean SID for CP and AA in wheat are similar to previously reported percentages (Stein et al., 2010; NRC 2012). The mean SID of CP and Lys for all wheat samples was 87.2% and 76.8%, respectively. Lysine has the lowest SID of all AA which is consistent with previous literature. Among other cereal grains, wheat is higher in SID of CP and all indispensable AA compared to barley and sorghum. The SID of CP and AA in wheat is similar to corn, however, it has higher SID for Trp. Feed grade Trp is an expensive ingredient compared to other feed grade AA for swine. Therefore, less supplemental Trp is needed when feeding a wheat-based diet compared to a corn-based diet because of both AA content and digestibility. The order of first 5 limiting AA based on SID AA within wheat from collective literature is Trp, Met, Thr, Lys, and His.

**Figure 5.**
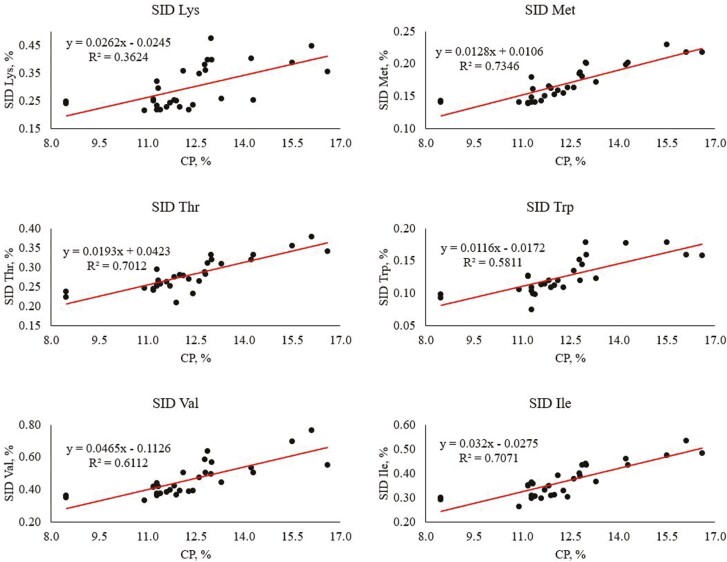
Simple linear regression to predict content of standardized ileal digestible (SID) Lys, Met, Thr, Trp, Val, and Ile in wheat in relation to the CP content (literature cited: Cervantes-Pahm et al., 2014b; Lee et al., 2020; McGhee and Stein, 2018; Oliveria et al., 2020; Rodriguez et al., 2020; Rosenfelder et al., 2015; Tan et al., 2021; Wang et al., 2018; Zhao et al., 2019. There were 30 samples for all AA except Trp which had 28 samples).

In general, as CP of wheat increased the SID of AA in wheat increased as well. Therefore, multiple regression models were developed to predict the content of SID AA within wheat in relation to the CP content (Table 8). These regression equations can be utilized to determine the approximate SID of each AA in a sample of wheat based on its CP concentration. As previously discussed, SID AA within wheat prediction models only provide an estimate based on CP and should be used with caution. Each CP regression coefficient was significant (*P *< 0.001) except for Pro and Tyr. To predict the relationship between CP and the content of SID Lys, a coefficient of 0.026 was found, meaning a 1% change in CP will result in a 0.026% change in SID Lys of wheat (Figure 5). The SID Lys had the poorest relationship with CP (lowest coefficient of determination) of all AA with significant *P* values (36.2%). Interestingly, it appears the regression equation may overestimate the SID Lys in wheat for samples that are under 12.5% CP and may underestimate the SID Lys in wheat for samples over 12.5% CP. Reasoning for this observation is unclear, but may be associated with the class of wheat, weather during growth, geographic location, and other external factors. For other indispensable AA, an increase in CP will have the greatest unit change on the SID Leu (0.074) and the least change on the SID Met (0.012). Leucine is also the indispensable AA that has the most variation in SID that is explained by CP (75.8%). For dispensable AA, an increase in CP will have the greatest unit change on the SID Glu (0.299) and the least change on the SID Ser (0.029). Glutamine is also the dispensable AA whose variation in SID is most explained by the CP (79.5%). The dispensable AA with the lowest coefficient of determination is Ala (50.5%).

**Table 8.** Simple linear regression to predict content of standardized ileal digestible (SID) AAs in relation to CP of wheat^1^

Item	*N* ^2^	Intercept	Slope (CP)	*R* ^2^	Standard error	*P*
Indispensable AA						
Arg	30	0.182	0.026	0.689	0.032	<0.001
His	30	−0.165	0.038	0.588	0.058	<0.001
Ile	30	−0.028	0.032	0.707	0.038	<0.001
Leu	29	−0.165	0.074	0.758	0.069	<0.001
Lys	30	−0.024	0.026	0.362	0.063	<0.001
Met	30	0.012	0.013	0.735	0.014	<0.001
Phe	30	−0.094	0.049	0.682	0.061	<0.001
Thr	30	0.042	0.019	0.701	0.023	<0.001
Trp	28	−0.017	0.012	0.581	0.018	<0.001
Val	30	−0.113	0.047	0.611	0.068	<0.001
Dispensable AA						
Ala	29	−0.077	0.037	0.505	0.066	<0.001
Asp	29	0.043	0.038	0.513	0.068	<0.001
Cys	29	−0.033	0.025	0.476	0.048	<0.001
Glu	29	−0.396	0.299	0.795	0.276	<0.001
Gly	28	0.038	0.032	0.534	0.049	<0.001
Pro	27	0.395	0.080	0.126	0.394	0.069
Ser	29	0.116	0.029	0.480	0.055	<0.001
Tyr	17	0.160	−0.002	0.001	0.107	0.926

^1^Literature cited: Cervantes-Pahm et al., 2014b; Lee et al., 2020; McGhee and Stein, 2018; Oliveria et al., 2020; Rodriguez et al., 2020; Rosenfelder et al., 2015; Tan et al., 2021; Wang et al., 2018; Zhao et al., 2019.

^2^Number of samples of wheat from collected publications utilized for mean values.

### Phosphorus

A major benefit to feeding wheat in diets is its concentration of P compared to corn and other cereal grains. Mean P was 0.27% summarized from this review, which is lower than previously reported values. Stein et al. (2010) reported average wheat P was 0.34% with a range of 0.23 to 0.38%. In addition, the NRC (2012) reported mean P for hard red and soft red wheat was 0.39 and 0.30%, respectively. It is unclear why mean P was lower in the current review but may be a consequence of less soil P from growing environmental concerns leading to less uptake by the plant. However, it is important to note the large standard deviation, with P ranging from 0.07 to 0.45% across all summarized literature. There were 5 wheat samples with P below 0.16% which resulted in a lower mean P than median P. The median P was 0.30% for all wheat samples. Removing the 5 low P wheat samples increased mean P to match median P of 0.30%. Although this is lower than previously reported, wheat is still higher in P than other cereal grains such as corn and sorghum.

Wheat contains intrinsic phytase that improves P digestibility in swine. In other cereal grains, a percentage of P is stored in the form of phytate-bound P which is largely unavailable to pigs because the amount of endogenous phytase in the small intestine is negligible (Eeckhout and DePaepe, 1994). Intrinsic phytase within wheat allows for increased release of phytate-bound P to improve P digestibility in pigs and reduce P supplementation in the diet (Poulsen et al., 2010). Brinch-Pedersen et al. (2014) reported the phytase activity of wheat was 1,637 FTU/kg whereas other cereal grains such as corn, rice, and sorghum were 190 FTU/kg or less. McGhee and Stein (2019) reported standardized total tract digestibility (STTD) of P was 36.6% in wheat-based diets compared to 24.9% in corn-based diets. When 1,000 FTU/kg of microbial phytase was added to the diets this resulted in an STTD of P at 57.6% and 62.5% for wheat and corn-based diets, respectively. The magnitude of P release is dependent on the cereal grain and amount of added phytase to the diets (Wu et al., 2004). Supplemental dietary P, often in the form of monocalcium or dicalcium phosphate, can be a costly component in swine diets. Therefore, high P and intrinsic phytase will allow for less P supplementation which can provide an economic advantage.

### Effect of Wheat in Swine Diets

Historically, research has suggested that wheat can be fed as the main cereal source in swine diets without affecting performance (Gill et al., 1966; Jensen et al., 1969). However, as genetics of both wheat and swine change to meet the goals of each industry, the response to wheat-based swine diets may change as well. In addition, different stages of production may elicit different responses when comparing wheat to other cereal grains.

### Nursery Pigs

Newly weaned pigs are particularly sensitive to ingredient changes and diet palatability (Wensley et al., 2021). Previous data suggests that wheat-based diets do not affect nursery pig performance when compared to corn-based diets (Rodriguez and Young, 1981). Mejicanos et al. (2017) and Bloxham et al. (2018) observed wheat-based diets did not affect nursery pig feed intake compared to corn-based diets. Both authors observed nursery pigs fed wheat-based diets had on average 6% improved feed efficiency compared to corn-based diets. In both studies, dietary treatments were balanced for energy and SID Lys; however, diet analysis indicated that the wheat-based diets had higher concentrations of GE and EE for the Mejicanos et al. (2017) and Bloxham et al. (2018) studies, respectively. Therefore, the improvements in feed efficiency associated with wheat-based diets may have been due to greater energy density of the diet. It is also possible better nutrient digestibility within wheat and the presence of gut promoting fibers could improve nutrient utilization (Bloxham et al. 2018; Bai et al., 2021). Gut promoting fibers in wheat may be a potential area of opportunity that is discussed more in depth in the wheat co-products portion of the review. Bloxham et al. (2018) also observed that ADG was highest when nursery pigs were fed a cereal blend of 60% corn and 40% wheat rather than a single cereal grain. Fouhse et al. (2016) and Zhou et al. (2016) observed nursery pigs fed barley-based diets resulted in improved ADG and feed efficiency compared to wheat-based diets. However, GE in barley diets from Zhou et al. (2016) was approximately 95 kcal/kg greater than wheat-based diets. Diets fed by Fouhse et al. (2016) were formulated to be isocaloric, but chemical analysis was not reported to confirm the formulation. It does appear differences in performance observed in various studies may be due to varying energy densities of the diet rather than a change in cereal grain.

Although there is little research comparing different classes of wheat, Jha et al. (2011) observed no differences in nursery pig growth performance between pigs fed white spring, red spring, hard red spring, durum, hard red winter, and hard white spring wheat of western Canada. Based on these results, the class of wheat should not affect nursery pig performance if nutritional differences among classes are considered.

### Finishing Pigs

Feeding wheat in finishing diets can provide certain formulation benefits as previously described, but it is also important to understand how wheat-based diets may influence performance and carcass characteristics. Similar to nursery pigs, Han et al. (2005) observed finishing pigs fed wheat-based diets had improved feed efficiency compared to corn-based diets, when beef tallow was added to the wheat-based diets to balance energy. Carr et al. (2005) observed pigs fed wheat-based diets had decreased ADFI, but no difference in feed efficiency than those fed corn-based diets. Chemical analysis revealed the wheat-based diet was 65 kcal/kg lower in ME than the corn-based diets, but still did not significantly influence ADG or feed efficiency. McConnell et al. (1975) observed growth performance between wheat-based diets and corn-based diets was comparable. For carcass characteristics, McConnell et al. (1975) and Carr et al. (2005) observed no differences when comparing pigs fed wheat-based diets to corn- and barley-based diets. In contrast, although Han et al. (2005) did not observe any differences in ADG, pigs fed wheat-based diets had lighter slaughter and carcass weights with all other carcass characteristics unaffected compared to pigs fed corn-based diets. In addition, Wang et al. (2023a) observed that although increasing levels of wheat in finishing pig diets did not affect growth performance, it significantly enhanced lightness, redness, and yellowness in the longissimus dorsi muscle. For fat color, Han et al. (2005) observed backfat lightness, redness, and yellowness was not affected when feeding finishing pigs either a corn or wheat-based diet. Although research shows some variable results, most response criteria in the literature were similar to feeding wheat compared to other cereal grains. Therefore, wheat is a suitable cereal grain for finishing pig performance while the effects on carcass characteristics remains variable and should be considered when implementing a change in cereal grains.

In addition to growth performance and carcass characteristics, wheat-based diets have also been observed to reduce fecal N excretion compared to corn-, barley-, and sorghum-based diets (Leek et al., 2007; Pan and An, 2020). However, Leek et al. (2007) observed urinary N excretion was greater and total N retention was decreased in pigs fed wheat-based diets compared to corn and barley. If N excretion is an environmental concern, feeding wheat-based diets can increase N excretion mainly driven by urinary N losses. However, enzymes can be implemented into diets to mitigate this issue.

Research has also shown the class of wheat can affect performance in finishing pigs, in contrast to early findings in nursery pigs. Pigs fed hard red winter wheat had increased ADG and ADFI compared to pigs fed soft red winter wheat and no differences in carcass characteristics (De Jong et al., 2016). The authors speculated that soft red winter wheat may have been lower in energy than expected.

### Sows

For sow and litter performance, maximizing sow lactation feed intake is important for optimal milk production to support litter growth. Therefore, it is important to understand how wheat affects ADFI and subsequent performance compared to other cereal grains. Park et al. (2010) observed feeding wheat-based diets led to reduced ADFI, poorer sow body condition, reduced reproductive performance, increased wean-to-estrus interval, and poorer litter performance than corn-based diets even though diets were balanced for energy. The authors speculated that nutrient digestibility in corn was greater than wheat which resulted in the observations. Although corn and wheat-based diets were formulated to be isocaloric, the corn-based diets had 0.41% greater average fat content compared to wheat-based diets potentially leading to greater energy density associated with corn-based diets. Stein et al. (1999) observed wheat-based diet’s apparent total tract digestibility of CP was 16.2% units greater than corn-based diets for both gestating and lactating sows. The authors also observed sows fed wheat-based diets had 0.7 and 1.1 kg greater ADFI during lactation compared to corn and barley-based diets, respectively. Bryant et al. (1985) and Nyachoti et al. (2006) observed similar lactation performance in sows fed either wheat or corn-based diets. However, Bryant et al. (1985) observed sows fed wheat-based diets had decreased total born, live born, and litter birth weight compared to sows fed corn-based diets. Diets were formulated to be isocaloric and isonitrogenous, but wheat-based diets did have 2.2% greater CP and 36 kcal/kg lower GE. Although there is little research available on wheat-based diets in gestation, the greater fiber content associated with wheat may help to increase satiety compared to other cereal grains such as corn and sorghum. Overall, it appears wheat can be utilized effectively in sow diets if energy density is not reduced. To our knowledge, there are no studies comparing different classes of wheat in sow diets. The only study to report the class of wheat utilized was Bryant et al. (1985) where they utilized soft winter wheat.

Although there are some inconsistencies in the literature, wheat-based diets appear to be comparable to other cereal grains across all phases of production. Differences observed in the literature may be due to different energy densities of the diets rather than a change in cereal grain. Mycotoxin-contamination not reported by authors could also explain inconsistencies in the literature in relation to pig performance which is discussed later in this review. Based on current literature, the class of wheat has been shown to have an impact on finishing diets, but not nursery pigs. However, further research is warranted in this area.

### Processing and Pelleting

Determining the optimal particle size from grinding is important for ideal performance in pigs. DeJong et al. (2016) observed a linear improvement in feed efficiency when wheat particle size was reduced from 728 to 326 µm in finishing pigs. In contrast, Bao et al. (2016) observed no differences in feed efficiency with reducing wheat particle size from 670 to 330 µm in growing pigs. The authors did observe a quadratic effect with ADG initially increasing from 670 to 450 µm and then decreasing from 430 to 330 µm. Finer particle sizes are thought to lead to improvements in pig performance because of increased surface area and nutrient digestibility. Grinding wheat too fine has been theorized to cause flour accumulation which may lead to increased stomach keratinization and ulceration. However, Mavromichalis et al. (2000) suggested factors causing stress to finishing pigs is needed for ulceration and should not be blamed simply on grinding wheat too fine. Finely ground wheat does increase dust accumulation and requires more electrical power to reach a target particle size. Stein et al. (2010) recommended wheat particle size should be no less than 500 µm for pigs to avoid these potential issues.

Utilizing either a hammer mill or roller mill for wheat particle size reduction can influence the response in pigs. Acosta et al. (2020) observed nutrient digestibility for finishing pigs was best when wheat was ground to 500 µm utilizing a hammer mill. However, when utilizing a roller mill the most optimal particle size for finishing pigs was 700 µm for nutrient digestibility. In growing pigs, the authors observed the optimal particle size was 500 µm regardless of particle size reduction method. Traditionally, roller mills are recommended for reducing the particle size of wheat because of improved uniformity and fewer fines (Goodband et al., 1995). Choct et al. (2004) observed that diets with wheat ground through a hammer mill significantly improved feed efficiency and numerically improved ADG compared to pigs fed wheat-based diets ground through a roller mill. However, the roller mill diets had a larger average particle size than the hammer mill diets which could have resulted in the feed efficiency response. Regardless of the method to reduce particle size of wheat, grinding to small particle sizes will lead to more electrical consumption and lower throughput (Bao et al., 2016; De Jong et al., 2016). It is important to determine the optimal balance for performance, stomach integrity, and feed milling efficiency within a production system for the most economical outcome.

In addition to particle size and grinding method, bulk density should also be a concern especially when utilizing wheat co-products. As wheat co-product inclusion increases, bulk density of the diet decreases because of added fiber. The change in bulk density will be more substantial with high-fiber wheat co-products such as wheat bran and middlings which is discussed in the wheat co-products portion of the review. A low bulk density diet can be associated with increased gut fill, limiting pigs’ ability to consume enough feed to meet their energy demand. In addition, low bulk density diets can result in poor feed flowability potentially limiting feed accessibility and causing out-of-feed events. Bulk density of wheat middlings has been suggested as an indicator of assessing wheat middlings quality (Cromwell et al., 2000). Light bulk density wheat middlings are generally associated with more bran material whereas heavier bulk density wheat middlings are associated with more residual endosperm (Cromwell et al., 1992; Salyer et al., 2012).

A strategy to increase bulk density is pelleting swine diets. Pelleted diets will also improve palatability, reduce dust, reduce feed wastage, improve handling and transportation, and increase nutrient utilization (Lancheros et al., 2020). To achieve these benefits from pelleting, it is important to have high quality pellets for swine. Wheat-based diets are often associated with increased pellet durability because of its gluten and hemicellulose content which are able to bind particles together compared to other cereal grains such as corn (Behnke, 2019). Stevens (1987) and Abdollahi et al. (2010) observed wheat-based diets improved pellet durability index (PDI) by 6.3% and 2.2%, respectively, compared to corn-based diets. In contrast, Amerah et al. (2008) observed corn-based diets had increased PDI compared to wheat-based broiler diets. However, all treatments had a PDI of at least 91.5% indicating all had good quality pellets. Even if wheat is not fed as the main cereal grain for a swine ration, it can be used as a pelleting aid. Behnke (1990) and Moradi et al. (2018) observed PDI progressively increased by an average of 1.4%, 5.7%, and 8.38% when incorporating wheat from 0%, 5%, 10%, and 20%, respectively, in corn or sorghum-based diets. Research also suggests the class of wheat can also influence pellet durability. De Jong et al. (2016) and Carré et al. (2005) observed hard wheat classes lead to increased PDI compared to softer classes.

Wheat co-products that contain proportions of the endosperm are also an option to improve pellet durability of swine diets. The endosperm is where gluten-forming proteins are found which are responsible for improving pellet quality (Blancher et al., 2005). Therefore, wheat middlings, millrun, shorts, and red dog would be expected to improve pellet quality, whereas wheat bran would not. Wecker et al. (2018) observed 7.4%, 11.6%, and 14.4% improvements in PDI when incorporating 2.5%, 5%, and 10% of a pasta co-product mainly comprised of wheat flour into corn-based diets. Starch gelatinization of cooked wheat co-products improves intra-particle bonds on the surface of the pellet, but overcooking can damage starch particles and diminish pellet quality (Behnke, 2001). Wecker et al. (2018) also observed 10% inclusion of the pasta co-product resulted in significantly increased PDI compared to a commercial pellet binder. Similarly, Saleh et al. (2021) observed 5% inclusion of wheat middlings improved PDI by 2.7% in a corn-based diet. Based on current research, 10% wheat or wheat co-products, with the exception of wheat bran, can be used to increase pellet quality. Pellet quality will continue to improve with higher wheat inclusion, but improvements may be diminishing.

Overall, wheat can provide opportunities in swine diets because of its high concentration and digestibility of nutrients. Although wheat may have lower energy compared to some other cereal grains such as corn, wheat-based diets are high in CP, AA, and P compared to other cereal grains. All aspects will factor into the total diet cost to be able to make decisions on formulation strategies. Furthermore, pelleted swine diets give additional value to wheat compared to meal diets largely because of reduced fines and less feed wastage.

## Feeding Off-Quality Wheat

### Sprouted Wheat

Sprouted grains are seeds that have begun to germinate and are harvested before they grow into a plant (Benincasa et al., 2019). Wet weather conditions can lead to delayed harvest which can cause small grains to sprout after they mature (Thomason et al., 2019). Wheat is particularly sensitive to sprouting, specifically soft classes of wheat that are grown in damp regions with excess rain, fog, and humidity (Nagelkirk, 2017). Sprouted wheat is typically not suitable for the milling industry and is consequently sold at a discount and used in livestock diets (Marston and DeRouchey, 2004). In 2020, each percent increase in the proportion of sprouted wheat resulted in a discount of 0.04 U.S. dollars/bu ($1.13/m^3^) across all classes and subclasses of wheat (USDA, 2020).

In sprouted wheat, the activity of α-amylase increases which is responsible for starch breakdown (Benincasa et al., 2019). On average, α-amylase activity increases by 94% in sprouted wheat compared to non-sprouted wheat (Simsek et al., 2014). The starch in sprouted wheat is broken down into glucose which serves as the energy source for germination. As a result, sprouted wheat contains less total starch but more soluble starch than non-sprouted wheat. Świeca et al. (2017) observed that total starch content was reduced from 65.2% in wheat flour to 58.4% in sprouted wheat flour. However, the total proteins were not significantly different between sprouted and non-sprouted wheat flour. Ultimately, the effects on the nutrient composition are dependent on the proportion of sprouted vs. non-sprouted kernels (Benincasa et al., 2019). Therefore, proximate and starch analyses should be performed to confirm nutrient profiles for inclusion in swine diets.

Research evaluating sprouted wheat has focused on the percentage of sprouted to non-sprouted wheat and not the severity of sprouting. Murray and Huber (1968) observed weanling pigs fed increasing proportions of sprouted to non-sprouted wheat had similar ADG but poorer feed efficiency. The authors observed proportions of 20%, 40%, and 60% sprouted wheat resulted in 3.9%, 6.8%, and 7.8% poorer feed efficiency compared to pigs fed only non-sprouted wheat, respectively. In addition, Eide et al. (1979) observed pigs fed a proportion of 20% sprouted hard red spring wheat improved feed efficiency by 11% compared to a barley control diet with no effect on ADG. However, pigs fed a proportion of 40% sprouted hard red spring wheat had similar performance as the barley control diet. Therefore, the 20% sprouted hard red spring wheat diet still had greater energy density than the barley diet, while the 40% sprouted hard red spring diet was comparable to the barley diet. In addition, Eide et al. (1979) also observed pigs fed a proportion of 20% and 40% sprouted durum improved growth performance compared to the barley control diet, with performance being most improved in the 20% sprouted durum diet. The 20% sprouted durum diet improved ADG and feed efficiency by 17% and 13%, respectively, whereas the 40% sprouted durum diet improved ADG and feed efficiency by 4% and 11%, respectively. Therefore, as the proportion of sprouted to non-sprouted wheat increases, the energy value will decrease. However, sprouted wheat can still be fed effectively in swine diets if the energy density of the diet is considered. In addition, more severe sprouting will have a greater impact on energy and should be fed at lower proportions than moderately sprouted wheat.

Sprouted wheat can also have an impact on grinding characteristics. Particle size of sprouted wheat kernels will tend to be smaller than non-sprouted kernels. This is a result of more accumulation of fine particles and less coarse particles from grinding sprouted wheat (Fu et al., 2014; Dziki et al., 2015). Therefore, it may be beneficial to target a coarser particle size for sprouted wheat to reduce the number of fine particles. In addition, the energy requirements to obtain a specific particle size will be less when using sprouted wheat compared to non-sprouted wheat (Dziki and Lakowski, 2010). These modifications will result in more significant changes to the milling characteristics as severity of sprouting becomes greater.

If sprouted wheat is available and economically priced into a swine diet, it can be effectively utilized. Research suggests sprouted wheat contains lower concentrations of energy than non-sprouted wheat, therefore, depending on the proportion of sprouted to non-sprouted wheat, the energy value of wheat should be adjusted. The energy content of 10% to 40% sprouted wheat should be discounted by approximately 10%, whereas a proportion of sprouted wheat 40% or greater energy should be decreased by 15% to 20%. In addition, proximate analysis should be performed to account for any other nutrient changes from severe sprouting.

### Low-Test Weight Wheat

The test weight of wheat is a measure of grain density or the weight at a specific volume. Test weight in wheat is influenced by different classes/varieties, growing conditions, high moisture around harvest, insect damage, and sprout damage (Wang and Fu, 2020). Freezing when the wheat kernel is still developing is a major cause of low-test weight wheat (Jones et al., 2019). The test weight of wheat will affect its grade and ultimately its end use. Once test weight begins to fall below 60 lb/bu (772 kg/m^3^), the U.S. grade will progressively worsen (USDA, 2014). Although, hard red spring and white club wheat (a subclass of soft white wheat) may naturally have lighter test weights than other classes and subclasses.

The nutritive value of low-test weight wheat will be dependent on the severity of test weight reduction. Low-test weight wheat is generally characterized at or below 51 lb/bu (656 kg/m^3^; Jones et al., 2019). As wheat kernels develop, protein is deposited first followed by starch. Therefore, if development is interrupted, the result is an incomplete starch deposition and increased protein-to-starch ratio (Lalman and Highfill, 2002). Therefore, low-test weight wheat will have decreased energy density. Protein may also need to be adjusted because of the change in the protein-to-starch ratio, but data is not available to confirm this hypothesis. It is estimated the feeding value of wheat is 95% to that of corn when wheat test weight is between 45 and 51 lb/bu (579 and 656 kg/m^3^). Hines and Pollman (1982) observed that decreasing wheat test weight from 59 to 45 lb/bu (759 to 579 kg/m^3^) resulted in no differences in ADG, but 6% and 8% poorer feed efficiency for 51 and 45 lb/bu (656 and 579 kg/m^3^) wheat, respectively. Therefore, feeding value of wheat is likely dependent on the severity of test weight reduction.

### Mycotoxin-Contaminated Wheat

Mycotoxins are naturally occurring toxins produced from certain molds and fungi found on a variety of different crops. In wheat, the most common mycotoxins are classified into 1 of 7 groups which include Aflatoxins, A-Trichothecenes, B-Trichothecenes, Fumonisins (FUM), Zearalenone (ZEN), Ochratoxin A (OTA), and ergot alkaloids (DSM, 2022). DON is a B-Trichothecene which is most common in wheat. A 10-year survey conducted by Gruber-Dorninger et al. (2019) reported DON was present in 65% of wheat samples with a median concentration of 0.37 mg/kg. The authors also reported 28% of wheat samples contained both DON and ZEN. In addition, ZEN, FUM, aflatoxin, and OTA were present in 33%, 14%, 10%, and 9% of wheat samples, respectively. The response in pigs to feeding wheat and wheat co-products containing mycotoxins will depend on the type of mycotoxin, concentration, presence of multiple mycotoxins, and stage of pig production.

The U.S. Food and Drug Administration (2010) guidance level for maximum DON contamination in wheat and wheat co-products intended for swine feeding is 5 mg/kg and that this grain does not exceed 20% of the diet corresponding to 1 mg/kg of DON in the complete diet. A simple linear regression was developed from collected publications to determine the effects of increasing DON level of the diet from DON-contaminated wheat on pig performance. Only diets with DON contamination above 1 mg/kg were included in the regression analysis based on the guidance levels of the FDA. For nursery pigs, each 1 mg/kg increase in DON-contaminated wheat-based diet resulted in an 11% and 6% decrease in ADG and ADFI, respectively. For finishing pigs, each 1 mg/kg increase in a DON-contaminated wheat-based diet resulted in a 2.6% and 2.7% decrease in ADG and ADFI, respectively. Therefore, these data would indicate that finishing pigs can tolerate higher DON levels in wheat-based diets compared to nursery pigs. However, the finishing pig studies had longer feeding durations of DON-contaminated wheat diets than nursery diets. Therefore, pigs may have been able to adjust to DON-contaminated wheat resulting in smaller changes in growth performance which has been observed in corn-based diets (Becker et al., 2022). It is unclear why pig performance is more adversely affected early in feeding DON-contaminated diets rather than later, but it may be associated with initial immune activation increasing the pigs maintenance requirement.

Although less prevalent, other mycotoxins can infect wheat grains and influence pig performance. Zearalenone is of most concern for breeding swine because of substantial effects on reproduction (Etienne and Jemmali, 1982; Jiang et al., 2011). Alm et al. (2006) observed oocyte degradation and reduced meiotic competence from feeding 0.235 mg/kg ZEN-contaminated wheat to 6-m-old gilts. Thus, feeding ZEN-contaminated wheat should not be fed in diets for the breeding herd. High OTA levels can decrease pig performance and also accumulate in the tissues because of its long biological half-life (Aoudia et al., 2009). Aoudia et al. (2009) observed pigs fed 0.12 mg/kg OTA-contaminated wheat-based diets led to reduced growth performance and increased concentrations of OTA in plasma, kidney, and liver up to 4 weeks after diets were fed. Recently, contamination of FUM in cereal grains has been increasing (Rao et al., 2020). Bracarense et al. (2012) observed wheat-based diets contaminated with a combination of FUM and DON negatively affected intestinal morphology and cytokine expression, but wheat diets contaminated with FUM alone provided similar results to feeding the uncontaminated wheat diet. Ergot alkaloids can also be prevalent in wheat during years of heavy rainfall and wet soil during the flowering stage (Krskaab and Crews, 2008). Wheat is the second most susceptible cereal grain to ergot contamination, only preceded by rye (Coufal-Majewski et al., 2016). Oresanya et al. (2003) observed that increasing ergot-contaminated wheat-based diets up to 20.8 mg/kg linearly decreased BW by 24%, ADG by 37%, ADFI by 10%, and G:F by 29% in nursery pigs. Most of the previously mentioned mycotoxins have guidance levels set by either the U.S. Food and Drug Administration (2000) or European Commission (2006) that should be referenced when feeding mycotoxin-contaminated wheat. However, aflatoxin is the only mycotoxin that has a mandatory action level set by the FDA. The FDA requires wheat or wheat co-products intended for swine diets to contain less than 20 µg/kg and it cannot be blended to decrease the concentration (FDA, 2000). Therefore, any wheat or wheat co-product that exceeds this action level must not be used or sold.

Wheat co-products are also a potential opportunity for mycotoxin-contamination. A survey by Gott et al. (2019) revealed that 91.8% of wheat middling samples contained at least one mycotoxin with DON and ergot alkaloids most frequently found. Among positive wheat middling samples, the mean concentration of DON and ergot alkaloids was 1.9 and 0.5 mg/kg, respectively. Therefore, mycotoxin-contamination of wheat co-products should not be overlooked as a potential source of contamination of the complete diet.

Mycotoxin-contaminated wheat can lead to economic losses in pig production depending on the class of mycotoxins and their concentration. To prevent mycotoxin-contamination after harvest, ensure proper storage of wheat to avoid moisture accumulation. Organic acids can be used to prevent mold growth. Propionic, acetic, and formic acids have been observed to have high inhibition against certain molds, whereas citric acid may help to a lesser degree (Hassan et al., 2012). If mycotoxins are found to be present in wheat, there are several methods to reduce its harmful effects. Contaminated wheat grains can be cleaned to remove fines and damaged kernels that often contain higher mycotoxin concentrations. Optical NIRS grain sorters are an effective method to lower mycotoxin levels in contaminated wheat. Commercial mycotoxin mitigants can be used to lessen the negative effects on pig growth. Blend contaminated wheat with uncontaminated wheat at a ratio that will fall below the allowance levels established by the FDA or European Commission when allowed. Mycotoxin-contaminated wheat should only be used as a partial grain source in swine diets. The last precaution would be to avoid using mycotoxin-contaminated wheat for nursery and breeding herd diets and fed only to finishing pigs.

Sprouting, low-test weight, and mycotoxin-contamination can be caused by many factors, with high-moisture field conditions most frequently associated. Therefore, wheat produced in high-moisture environments is more likely to become off-quality. While sprouted and low-test weight wheat can still have a negative effect on performance, research suggests they can be fed effectively in swine diets as long as energy is adjusted accordingly depending on the severity of damage and proportion fed. High levels of mycotoxin-contamination would likely lead to more severe reductions in performance of pigs compared to sprouted and low-test weight wheat. Therefore, mycotoxin-contamination should be of most importance when evaluating off-quality wheat. The level and type of mycotoxin present in wheat will ultimately determine its application in swine diets.

## Feeding Wheat Co-Products

### Co-Product Production

**Figure 6.**
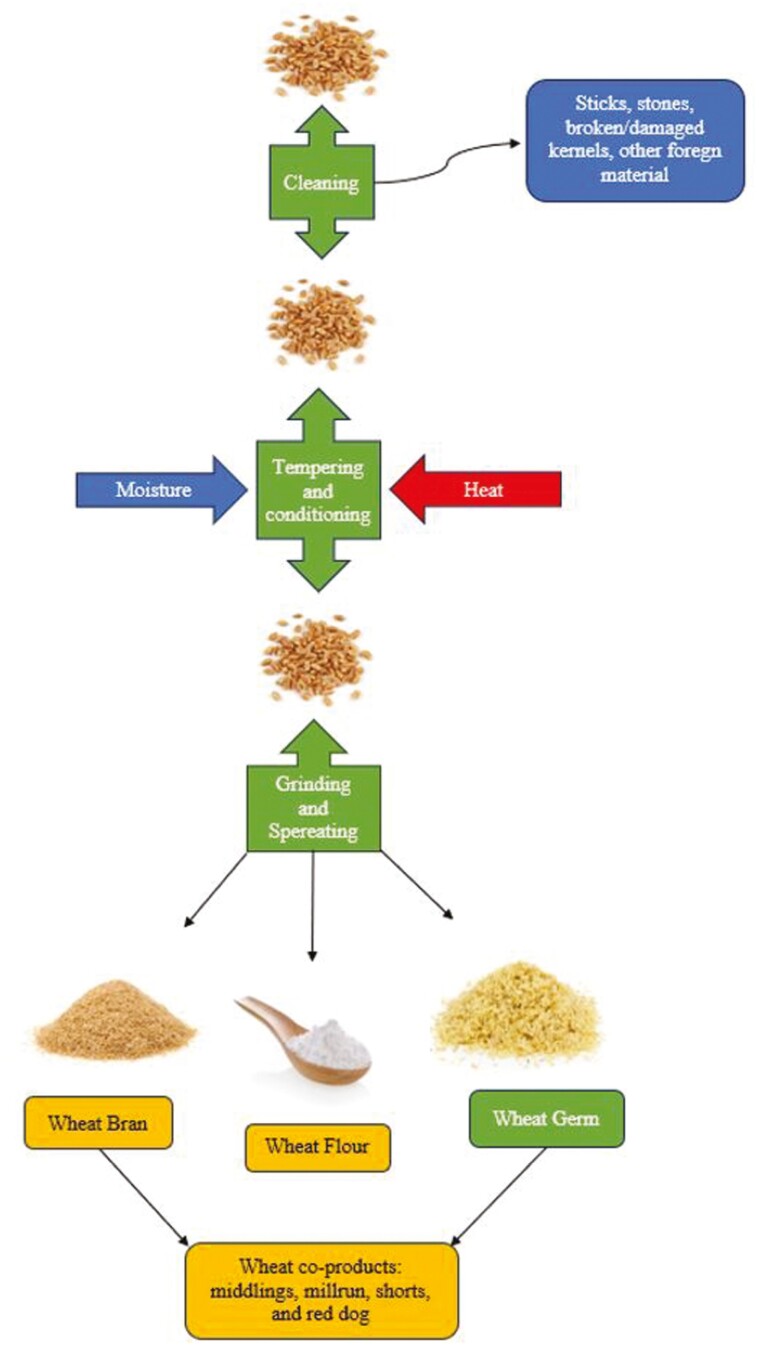
Wheat milling flow diagram.

During the milling process to produce wheat flour, the wheat kernel is separated to obtain the nutritional-rich components. The wheat kernel is mainly comprised of bran (protective outer layer), endosperm (nutritional reserves for germination), and germ (reproductive component; Campbell, 2007). The endosperm is rich in starch and protein that is extracted for flour milling. Once the endosperm is separated, the germ, bran, and some residual endosperm components remain, which make up the various wheat co-products (Figure 6). Wheat bran is defined as the coarse outer covering of the wheat kernel as separated from cleaned and scoured wheat in the usual process of commercial milling (AAFCO, 2023). Other wheat co-products such as wheat middlings (often referred to as wheat midds), wheat shorts, wheat millrun, and wheat red dog are produced when wheat bran is combined with wheat germ, wheat flour, and some offal from the “tail of the mill” (AAFCO, 2023). However, depending on the combination and concentrations of these fractions the co-product classification changes (Huang et al., 2012). The Association of American Feed Control Officials (2023) classifies each co-product based on crude fiber (CF) content in which wheat middlings, millrun, shorts, and red dog must contain no more than 11%, 9.5%, 7%, and 4% CF, respectively. The definition provided by AAFCO (2023) for wheat bran does not provide a CF classification as it does with the other co-products, but it can be assumed that CF should be greater than 11% CF because it only contains the outer covering of the wheat kernel and no germ or endosperm components.

Although each wheat co-product classification is outlined by AAFCO (2023), there are discrepancies in terminology depending on geographical location. Wheat millrun is commonly characterized in Canadian literature, but little is available outside of that region. High variability is often associated with wheat millrun contributing to its classification difficulty. Yarlagadda and Lee (2008) utilized the terms wheat millrun and wheat midds interchangeably when referring to leftover materials after flour extraction. Similarly, there appear to be some discrepancies between wheat middlings and bran between North America and Europe (Espinosa et al., 2024). The reclassification process of some sources of co-products was based AAFCO (2023) definitions of wheat co-products and nutrient outliers to correct these discrepancies in the current review.

Other wheat co-products exist outside of the flour milling industry such as wheat products from biofuel production. A review conducted by Rosenfelder et al. (2013) discusses additional wheat co-products such as wheat dried distillers grains. Wheat flour co-products are commonly characterized in the literature focused on swine, but organization and discussion of their nutritional differences and application in swine diets is warranted.

### Nutrient Concentrations

Wheat co-product classification is based on the amount of bran, germ, and residual endosperm fractions present (Sarfaraz et al., 2017). Transitioning from wheat bran to middlings to millrun to shorts to red dog is associated with less bran and more germ and flour fractions. Starch concentration increases from each co-product to the next which results in similar reductions in other nutrient values, particularly fiber (Table 9).

**Table 9.** Nutrient and AA composition of wheat co-products (as-fed basis)^1^

Item	Wheat bran		Wheat middlings		Wheat millrun		Wheat shorts		Wheat red dog	
	*N* ^2^	Mean ± SD	*N* ^2^	Mean ± SD	*N* ^2^	Mean ± SD	*N* ^2^	Mean ± SD	*N* ^2^	Mean ± SD
Nutrient composition, %										
DM	24	89.2 ± 1.86	47	89.1 ± 1.41	4	89.2 ± 0.87	7	89.9 ± 2.96	7	90.8 ± 2.07
CP	26	16.0 ± 0.90	47	16.5 ± 1.77	4	16.6 ± 0.65	20	16.3 ± 2.04	19	13.8 ± 1.21
EE	19	3.1 ± 0.94	44	4.0 ± 1.12	4	3.9 ± 0.91	14	3.1 ± 0.72	14	2.4 ± 0.61
Ash	20	5.2 ± 0.70	37	5.2 ± 0.82	4	5.5 ± 0.28	14	3.3 ± 1.08	14	2.7 ± 1.79
CF	5	10.2 ± 1.23	16	8.4 ± 0.67	4	8.9 ± 1.36	17	5.3 ± 0.94	16	2.6 ± 1.04
ADF	20	11.3 ± 1.63	33	10.5 ± 1.51	5	13.6 ± 2.10	21	6.3 ± 2.78	15	2.5 ± 1.05
NDF	21	39.4 ± 4.01	33	34.7 ± 4.20	5	34.4 ± 3.43	21	25.0 ± 5.99	15	11.6 ± 4.04
TDF	12	42.9 ± 4.35	17	37.8 ± 4.24	1	38.0	1	19.3	1	13.9
IDF	12	39.6 ± 4.46	16	34.7 ± 4.72	1	35.5	1	15.2	1	12.8
SDF	12	3.2 ± 0.83	16	2.9 ± 1.28	1	2.4	1	4.1	1	1.1
Ca	7	0.11 ± 0.034	35	0.14 ± 0.127	3	0.14 ± 0.051	16	0.05 ± 0.029	18	0.16 ± 0.252
P	8	0.99 ± 0.135	35	1.05 ± 0.169	3	0.97 ± 0.136	16	0.40 ± 0.279	18	0.30 ± 0.092
Starch	11	14.5 ± 3.29	30	20.3 ± 3.21	2	20.3 ± 7.19	11	33.3 ± 7.94	13	54.9 ± 8.89
Indispensable AA, %										
Arg	8	1.03 ± 0.139	29	1.07 ± 0.073	1	1.09	8	0.93 ± 0.149	7	0.68 ± 0.131
His	8	0.45 ± 0.074	29	0.43 ± 0.028	1	0.42	8	0.39 ± 0.041	7	0.32 ± 0.030
Ile	8	0.49 ± 0.039	29	0.52 ± 0.038	1	0.52	8	0.49 ± 0.037	7	0.44 ± 0.063
Leu	8	0.94 ± 0.096	29	1.01 ± 0.070	1	1.01	8	0.99 ± 0.048	7	0.91 ± 0.075
Lys	8	0.62 ± 0.070	29	0.70 ± 0.049	1	0.74	8	0.59 ± 0.078	7	0.45 ± 0.110
Met	8	0.23 ± 0.021	29	0.24 ± 0.020	1	0.23	8	0.25 ± 0.028	7	0.24 ± 0.059
Phe	8	0.60 ± 0.095	29	0.64 ± 0.048	1	0.65	8	0.65 ± 0.071	7	0.62 ± 0.076
Thr	8	0.49 ± 0.053	29	0.52 ± 0.034	1	0.52	8	0.51 ± 0.062	7	0.41 ± 0.072
Trp	5	0.18 ± 0.049	29	0.17 ± 0.030	1	0.19	6	0.20 ± 0.031	7	0.16 ± 0.015
Val	8	0.72 ± 0.071	29	0.76 ± 0.056	1	0.75	8	0.73 ± 0.084	7	0.60 ± 0.064
Dispensable AA, %										
Ala	5	0.79 ± 0.071	27	0.77 ± 0.052	1	0.79	2	0.85 ± 0.088	1	0.63
Asp	5	1.12 ± 0.052	27	1.13 ± 0.084	1	1.15	2	1.24 ± 0.186	1	0.98
Cys	5	0.36 ± 0.051	27	0.32 ± 0.016	1	0.30	2	0.30 ± 0.193	1	0.29
Glu	5	2.85 ± 0.296	27	2.96 ± 0.191	1	2.72	2	2.84 ± 0.327	1	3.58
Gly	5	0.85 ± 0.029	27	0.84 ± 0.041	1	0.85	2	0.84 ± 0.023	1	0.69
Pro	3	0.92 ± 0.058	17	0.95 ± 0.106	1	0.93	1	0.92	1	1.13
Ser	5	0.61 ± 0.049	27	0.59 ± 0.065	1	0.59	2	0.69 ± 0.095	1	0.58
Tyr	5	0.37 ± 0.073	25	0.40 ± 0.051	1	0.46	2	0.36 ± 0.134	1	0.43

^1^Literature cited: Abelilla and Stein, 2019; Acosta et al., 2017; Adedokun and Adeola, 2020; Adedokun et al., 2019; Berrocoso et al., 2015; Casas et al., 2018; Chee et al., 2005; De Jong et al., 2014; Eklund et al., 2014; Espinosa et al., 2024; Franceschina et al., 2015; He et al., 2021; Huang et al., 2013; Huang et al., 2014; Huang et al., 2015; Jaworski et al., 2015; Jaworski et al., 2016; Jaworski and Stein, 2017; Jha et al., 2012; Kim et al., 2022; Kraler et al., 2014; Lee et al., 2020; Lee et al., 2022; Lowell et al., 2015; Lyu et al., 2023; Navarro et al., 2018; Nortey et al., 2008; Oliviera et al., 2020; Oliviera et al., 2022; Poulsen and Blaabjerg, 2016; Salyer et al., 2012; She et al., 2015; Son et al., 2023; Stewart et al., 2013; Trindade Neto et al., 2020; Wesendonck et al., 2013; Wu et al., 2016; Zhang et al., 2020; Zhao et al., 2018a, 2018b; Zhao et al., 2020.

^2^The number of wheat co-product samples from collected publications.

For direct comparisons, wheat middlings are slightly greater in CP, EE, Ca, P, and starch than wheat bran. Wheat bran mostly consists of the fibrous outer covering of the wheat kernel which explains its high fiber and low starch content. The concentration of starch in wheat bran from the current review is lower than the NRC (2012), which is likely due to recent advances in starch extraction during flour milling (Jaworski et al., 2016). In the literature, wheat middlings and millrun were very similar in their mean nutrient values. The similarities may be a result of terminology differences from geographical locations and the 2 co-products may be nutritionally equivalent. Wheat shorts had lower nutrient concentrations in almost all categories compared to wheat middlings and millrun, but higher in starch content. Similarly, wheat red dog had lower nutrient concentrations in almost all categories compared to wheat shorts, but higher in starch content. To our knowledge, this is the first review to summarize wheat shorts and red dog nutrient values based on current literature. A review by Rosenfelder et al. (2013) only characterized wheat bran and middlings, and the NRC (2012) only reports one citation for wheat shorts.

For AA profile levels, wheat middlings, shorts, and millrun generally had higher levels than wheat bran and red dog. This would be explained by those having higher CP percentages. Wheat middlings mean values were similar to observations for wheat millrun; however, only one journal article was obtained with the AA profile of wheat millrun. The Lys concentration of the various co-products was 0.74%, 0.70%, 0.62%, 0.59%, and 0.45% for millrun, middlings, bran, shorts, and red dog, respectively. When compared to other literature, wheat middlings AA profile is similar to values reported by the NRC (2012).

### SID of CP and AAs

To our knowledge, there is no published literature characterizing the SID of CP and AA for wheat millrun. We speculate that SID of CP and AA in wheat millrun is similar to wheat middlings because of their similarities in nutrient and AA content. The SID of CP for wheat bran, middlings, shorts, and red dog is 67.5%, 70.6%, 85.9%, and 88.1%, respectively (Table 10). The SID of Lys for wheat bran, middlings, shorts, and red dog is 69.3%, 70.2%, 87.9%, and 83.1%, respectively. Ten wheat middlings samples from Casas and Stein (2017) were removed from mean SID of CP and AA summarization because the author’s reported samples were heat damaged from processing. Lysine is particularly sensitive to heat damage because of its free amino group which easily reacts with a reducing sugar to form Maillard reaction products and becomes unavailable to the pigs (Almeida et al., 2013). Therefore, processing of wheat co-products should be monitored to avoid heat damage and negatively influence the digestibility of Lys and other AA in the co-products.

**Table 10.** SID of CP and AA in wheat co-products^1^

Item	Wheat bran		Wheat middlings^2^		Wheat middlings^3^		Wheat shorts		Wheat red dog	
	*N* ^4^	Mean ± SD	*N* ^4^	Mean ± SD	*N* ^4^	Mean ± SD	*N* ^4^	Mean ± SD	*N* ^4^	Mean ± SD
SID of CP	3	67.5 ± 5.83	11	70.6 ± 6.07	10	61.5	7	85.9 ± 10.52	6	88.1 ± 4.78
SID of Indispensable AA, %										
Arg	5	83.0 ± 6.55	17	83.3 ± 6.06	10	81.4	7	91.8 ± 5.30	6	92.1 ± 3.01
His	5	76.8 ± 9.59	17	80.0 ± 2.61	10	77.7	5	93.9 ± 1.63	6	89.8 ± 7.34
Ile	5	73.9 ± 5.11	17	74.9 ± 4.39	10	69.4	7	86.1 ± 11.05	6	89.7 ± 3.20
Leu	5	76.3 ± 5.38	17	78.5 ± 4.12	10	72.5	7	90.9 ± 5.17	6	91.7 ± 3.76
Lys	5	69.3 ± 9.61	17	70.2 ± 7.55	10	45.9	7	87.9 ± 4.20	6	83.1 ± 6.14
Met	5	76.9 ± 7.83	17	79.8 ± 4.51	10	73.6	7	91.3 ± 3.03	6	93.0 ± 1.57
Phe	5	75.9 ± 6.99	17	79.3 ± 3.63	10	69.8	5	94.2 ± 1.66	6	92.4 ± 3.51
Thr	5	68.1 ± 9.35	17	70.6 ± 4.53	10	62.7	7	86.7 ± 5.53	6	86.5 ± 2.24
Trp	2	66.1 ± 18.24	17	77.6 ± 7.27	10	70.5	5	89.0 ± 4.07	6	89.2 ± 1.28
Val	5	73.7 ± 6.00	17	75.2 ± 5.70	10	63.7	7	90.1 ± 4.62	6	86.8 ± 7.42
SID of Dispensable AA, %										
Ala	4	69.0 ± 6.30	15	68.2 ± 6.16	10	54.8	0	—	1	77.5
Asp	4	68.9 ± 3.32	15	72.1 ± 4.47	10	66.4	0	—	1	77.1
Cys	3	73.3 ± 1.86	15	75.6 ± 3.75	10	73.7	2	82.5 ± 0.71	1	57.4
Glu	4	82.7 ± 3.68	15	86.0 ± 2.84	10	81.8	0	—	1	91.9
Gly	3	71.9 ± 6.01	15	66.5 ± 8.30	0	—	0	—	0	—
Pro	3	85.8 ± 13.49	s2	120.5 ± 9.12	0	—	0	—	0	—
Ser	4	77.4 ± 3.50	15	76.2 ± 4.83	10	72.5	0	—	1	74.9
Tyr	4	75.8 ± 4.43	13	76.0 ± 4.94	10	68.4	0	---	1	85.5

^1^ Adedokun et al., 2019; Casas and Stein, 2017; Eklund et al., 2014; Espinosa et al., 2024; Huang et al., 2012; Huang et al., 2014; Jaworski et al., 2016; Koo et al., 2017; Lee et al., 2020; Lee et al., 2021; Libao-Mercado et al., 2006; Lyu et al., 2020; Lyu et al., 2023; Trindade Neto; Oliviera et al., 2020; Woyengo and Zijlstra, 2021; Zhao et al., 2018b. There were no journal articles with SID values for wheat millrun.

^2^Nonheat damaged wheat middlings samples.

^3^Heat damaged wheat middlings samples from Casa and Stein, 2018.

^4^The number of wheat co-product samples from collected publications.

There appears to be a clear divide between SID of CP and AA of wheat bran/middlings and wheat shorts/red dog. Wheat bran and middlings have lower SID percentages than wheat shorts and red dog. The total SID of all indispensable AA for wheat bran and middlings was 74.0% and 76.9%, respectively, whereas the total SID of all indispensable AA for wheat shorts and red dog was 90.2% and 89.4%, respectively. Therefore, including wheat shorts or red dog will result in better digestible CP and AA in swine diets than wheat bran or middlings.

Wheat middlings had greater SID values of CP and all indispensable AA compared to wheat bran. When comparing wheat shorts and red dog, SID of CP was greater for wheat red dog than shorts. Wheat red dog was also greater in SID of Arg, Ile, Leu, Met, and Trp while wheat shorts was greater in SID of His, Lys, Phe, Thr, and Val. However, the greatest difference between the co-products for SID of all indispensable AA was only 4.8% units observed for Lys. There was very little information from publications collected on the SID of dispensable AA for wheat shorts and red dog.

While wheat co-products are lower in SID of CP and AA compared to wheat grain because of higher fiber, they still have a higher concentration of total CP and AA. This allows the opportunity to add more feed grade Lys and other AA because CP needed for non-essential AA production is met utilizing wheat co-products. Overall, wheat co-products may have lower SID of CP and AA compared to the wheat grain they originated from, but they are still a low-cost ingredient with relatively high CP and AA.

### Phosphorus Concentration

Wheat co-products are excellent sources of P for swine diets. Wheat bran, middlings, and millrun P level were 0.99%, 1.05%, and 0.97%, respectively. Wheat bran, middlings, and millrun are at least 0.37% higher in P compared to other common co-products such as bakery meal, corn dried distiller grains, oat groats, and soybean hulls (NRC, 2012). Wheat is already a high P cereal grain, but when flour is extracted from wheat during the milling process P becomes more concentrated in the co-products resulting in increased P. The P concentration of wheat bran and middlings from the current review is similar to values reported by NRC (2012). The highest concentrations of intrinsic phytase are typically found in the bran of the wheat kernel (Cornell, 2012; Katamadze et al., 2023). Therefore, wheat bran and other co-products with high proportions of bran will have high intrinsic phytase activity. Feeding wheat bran, middlings, or millrun in swine diets will result in less needed supplemental inorganic P such as monocalcium or dicalcium phosphate and can reduce diet costs. Wheat shorts and red dog contain 0.40% and 0.30% P, respectively, which is similar to conventional wheat.

### Energy Concentration

The energy concentrations of wheat co-products are dependent on the level of starch and overall fiber (Huang et al., 2014; Lyu et al., 2023). Across all co-products, wheat bran had the lowest mean DE and ME values of 2,796 and 2,636 kcal/kg DM, respectively (Table 11). Wheat red dog was highest among co-products with average DE and ME of 3,665 and 3,473 kcal/kg DM, respectively. There was only a single publication from Woyengo and Ziljstra (2021) that reported DE and ME of wheat millrun. Therefore, no mean energy values were developed for wheat millrun, but we speculate energy values would be similar to wheat middlings because of similarities in nutrient profile. On average, mean ME across all co-products was determined to be 95% of mean DE in the current review.

**Table 11.** Energy content of wheat co-products^1^

Item	Wheat bran		Wheat middlings		Wheat shorts		Wheat red dog	
	*N* ^2^	Mean ± SD	*N* ^2^	Mean ± SD	*N* ^2^	Mean ± SD	*N* ^2^	Mean ± SD
Energy content (kcal/kg DM)								
GE	11	4,551 ± 67.5	20	4,468 ± 67.3	9	4,426 ± 286.7	10	4,343 ± 175.0
DE	12	2,796 ± 236.9	21	2,982 ± 141.2	9	3,344 ± 234.7	10	3,665 ± 238.1
ME	12	2,636 ± 219.3	20	2,872 ± 145.1	9	3,152 ± 185.6	10	3,473 ± 170.9
Calculated NE^3^	10	1,950 ± 179.7	11	2,085 ± 111.7	9	2,343 ± 163.8	10	2,578 ± 170.9

^1^ Adedokun et al., 2019; Casas et al., 2018; Espinosa et al., 2024; Huang et al., 2012; Huang et al., 2014; Jaworski et al., 2016; Kim et al., 2022; Lyu et al., 2020; Lyu et al., 2023; Stewart et al., 2013; Woyengo and Zijlstra, 2021.

^2^The number of wheat co-product samples from collected publications.

^3^Calculated using equations 1 to 8 from NRC (2012). Net energy (kcal/kg DM) = (0.700 × DE) + (1.61 × EE) + (0.48 × Starch) − (0.91 × CP) − (0.87 × ADF).

When comparing mean energy values of wheat bran to middlings from publications collected, the mean difference in DE was 186 kcal/kg, whereas the difference in ME was 236 kcal/kg. This is likely due to the higher CP, starch, and fat associated with wheat middlings in combination with lower fiber content. In contrast, the difference in DE was 362 kcal/kg when comparing wheat middlings and shorts mean energy values, whereas the difference in ME was 280 kcal/kg. This again could be a result of nutrient differences between the 2 co-products, but also wheat shorts may have a slower passage rate from greater soluble fiber allowing for more nutrient absorption and less excretion in the feces than middlings (Wenk, 2001). When comparing wheat shorts and red dog, the difference between mean DE and ME for both was 321 kcal/kg.

In addition, reported DE, EE, starch, CP, and ADF from each publication were utilized to calculate NE of each co-product (equations 1 to 8; NRC, 2012). Higher DE, EE, and starch within a wheat co-product sample would correlate to greater NE values, whereas greater CP and ADF would correlate to lower NE values. Calculated NE of wheat co-product samples followed a similar pattern as mean DE and ME with bran being the lowest and red dog being the highest. On average, mean calculated NE across all co-products was 74% of ME. Average GE was similar across wheat bran, middlings, shorts, and red dog with an average value of 4,451 kcal/kg DM and a range of 208 kcal/kg.

### Co-Product Influence on Growth Performance

The optimal wheat co-product level in a swine diet is a balance between diet cost, ingredient consistency, and growth performance. Co-products are often variable in nutrient concentration that may lead to variation in pig performance. The addition of fibrous wheat co-products may require feeder adjustments because of potential feed flowability issues. To have increased confidence in the co-product being evaluated or used, regular chemical analysis (moisture, CP, fiber [CF, ADF, and NDF]) is recommended to confirm the nutrient profile. Starch analysis may also be warranted particularly for wheat shorts and red dog because of their high concentrations compared to the other wheat co-products. As previously described, the levels of fiber, protein, starch, and other nutrients will impact how the co-product will optimize in inclusion rate and stage of production in a swine diet.

The majority of literature that focuses on pig performance from feeding wheat co-products consists of wheat bran and wheat middlings. Most wheat bran research has been conducted in nursery pigs to improve gastrointestinal health based on its fiber content. Wheat middlings research has been conducted in both nursery pigs and finishing pigs. Its application in finishing diets has gained attention because of it being a more economical source of nutrients compared to the higher cost of grain and intact protein ingredients commonly used in swine diets. Wheat millrun has literature in both nursery and finishing pigs, however, it is less prevalent than wheat bran and middlings. There is little research available on wheat shorts and published studies that are available were conducted prior to 1982. To our knowledge, there are no published studies evaluating the effects of wheat red dog on pig performance which warrants future attention.

### Nursery Pigs

When nursery diets are formulated to equal energy levels, research indicates diets containing wheat bran can improve feed efficiency without dietary antibiotic use. Gasa et al. (2010), Molist et al. (2011), and Zhao et al. (2018b) observed nursery pigs fed wheat bran improved feed efficiency by 8.8%, 8.1%, and 6.1%, respectively, compared to a control diet without wheat bran. In addition, Gasa et al. (2010) also observed an increase in ADG from pigs fed wheat bran compared to a control diet. Gasa et al. (2010) and Molist et al. (2011) fed wheat bran at 4% of the diet, whereas Zhao et al. (2018b) fed 5% wheat bran. Improvements in feed efficiency may be a result of improved gastrointestinal health of young pigs which is discussed later in the review. However, across all 3 studies, chemical analysis showed wheat bran diets were greater in GE than control diets that likely contributed to the feed efficiency response. Batson et al. (2021) and Laskoski et al. (2021) observed growth performance was comparable between pigs fed a control diet and a diet with 4% wheat bran in non-isocaloric diets. Therefore, research has shown wheat bran can be included at 4% to 5% of the diet without negatively affecting nursery pig performance whether diets are isocaloric or not.

De Jong et al. (2014) evaluated the effects of added wheat middlings on nursery pig performance. In these studies, all diets were not formulated to be isocaloric. In summary of the 4 experiments, increasing added wheat middlings decreased growth performance with 5%, 10%, 15%, and 20% wheat middlings decreasing ADG by 0.6%, 1.7%, 2.1%, and 5.0%, respectively, and ADFI by 1.4%, 1.7%, 2.6%, and 1.5%, respectively. Feed efficiency was relatively unaffected by increasing added wheat middlings until 20% inclusion which resulted in a 4.6% reduction in G:F. Shaw et al. (2002) also fed 10% wheat middlings to nursery pigs and observed similar reductions in growth performance as De Jong et al. (2014). However, Shaw et al. (2002) diets were formulated to be isocaloric unlike De Jong et al. (2014).

Garcia et al. (2015) conducted a study with increasing wheat millrun in addition to increasing levels of canola oil to balance dietary energy. The authors reported increasing wheat millrun from 0% to 20% of the diet led to a linear improvement in feed efficiency. However, chemical analysis revealed energy density of the diet also increased, thus indicating millrun, canola oil, or both were undervalued in energy at formulation. However, if wheat millrun is added to a nursery diet without balancing energy it will likely lead to a similar response to wheat middlings because of their nutrient similarities. To our knowledge, there are no studies evaluating nursery pig performance feeding wheat shorts or red dog. However, these co-products are higher in energy and may be able to be incorporated with similar or less impact on performance.

### Finishing Pigs

Nursery pig’s appetite limits their ability to consume enough feed to meet their growth potential. However, finishing pig’s feed intake exceeds their ability to meet their growth potential. Therefore, finishing pig growth performance will likely be more severely affected with increasing levels of wheat co-products than in nursery pigs because of the reduction in energy density of the diet. Du et al. (2023) fed increasing wheat bran (0%, 7%, 14%, and 21%) to finishing pigs and observed significant reductions in ADG and ADFI. All 3 levels of wheat bran inclusion led to similar reductions in ADG and ADFI by approximately 20% and 15%, respectively. Anguita et al. (2007) also observed reductions in ADFI from feeding 10% wheat bran, but they also observed an improvement in feed efficiency. However, the authors added soy oil to balance energy which resulted in the wheat bran diet having 95 kcal/kg greater GE based on chemical analysis.

Similar to nursery pigs, as inclusion rate of wheat middlings increase, performance decreases because of reductions in energy density of the diet. Salyer et al. (2012) observed 10% and 20% added wheat middlings decreased ADG by 3.2% and 4.5%, respectively, and G:F by 2.7% and 4.2%, respectively, although a constant SID Lys:ME/Mcal ratio was maintained. In contrast, Shaw et al. (2002) and Wu et al. (2016) did not observe any growth differences from feeding wheat middlings at 30% and 15% of the diet, respectively, when energy was balanced. In non-isocaloric diets, Stewart et al. (2013) observed 30% added wheat middlings lead to 16.0% and 11.2% poorer ADG and feed efficiency, respectively, compared to a control diet. Salyer et al. (2012), Stewart et al. (2013), and Wu et al. (2016) also observed decreased carcass weight and yield from feeding diets containing wheat middlings, however, Shaw et al. (2002) did not observe any differences in carcass characteristics.

Wheat millrun appears to lead to similar reductions in performance as wheat middlings. Nortey et al. (2007) observed pigs fed 20% and 40% wheat millrun decreased ADG by 2.9% and 6.9%, respectively, and G:F by 4.9% and 7.3%, respectively, in energy-balanced diets. In non-isocaloric diets, Kpogo et al. (2021) observed 30% added wheat millrun reduced ADG and feed efficiency by 2.7% and 5.1%, respectively. The authors also reported decreased back fat depth from feeding 30% wheat millrun. However, unlike wheat middlings, the authors observed an increase in carcass yield with feeding wheat millrun.

Available research suggests wheat shorts can be fed at a higher inclusion compared to previously mentioned wheat co-products due to their higher energy. Young (1980) observed pigs fed 32.2% wheat shorts had comparable performance to pigs fed a control diet with no added wheat shorts in non-isocaloric diets. However, when wheat shorts were fed at 64.4% and 96.6% of the diet performance decreased. To our knowledge, there are no published growth performance studies on wheat red dog in finishing pigs.

### Sows

In sow diets, wheat co-product application may be most beneficial during gestation. Limit feeding during gestation can cause aggressive behavior because sows may be unfulfilled by the amount of feed they receive (Meunier-Salaün et al., 2001). Incorporating wheat co-products during gestation may help to increase satiety in sows to reduce aggressive behavior (Guillemet et al., 2007). In addition, wheat co-products can be fed in diets for replacement gilts to limit ADG and reduce feed cost until breeding ultimately lowering the cost of nonproductive days. Wheat co-product inclusion may be more challenging in lactating sow diets because of their lower energy content, but if fed at lower levels they may be able to be used without diminishing feed intake. Shang et al. (2019) observed sows fed wheat bran in late gestation (30% of the diet) and lactation (15% of the diet) led to a numeric increase in sow feed intake. In this study, the gestation diet containing wheat bran was lower in energy but fed to achieve equal DE intake across all treatments, whereas the lactation diets were balanced for energy and fed ad libitum. The authors also observed piglets from the sows fed wheat bran had numerically greater weaning weight and ADG. Renaudeau et al. (2003) observed sows fed lactation diets containing a combination of wheat bran and wheat middlings at 47.9% of the diet resulted in greater body weight and backfat loss, but piglets had increased ADG and weight at weaning even though diets were formulated to be 263 kcal/kg less than the control diet. The authors speculated the improved litter growth was because of increased milk production, but mechanisms behind increased milk production were unclear. Young and King (1981) observed sows fed diets completely comprised of wheat shorts during gestation had 1.2 more pigs weaned, but 0.08 kg lighter birth weight than sows fed a corn–soybean meal gestation diet. To our knowledge, there is no research evaluating wheat millrun or red dog in sow diets and the effect it has on sow and litter performance.

High-fiber wheat co-products such as wheat bran, middlings, and millrun can be fed in all stages of production, but performance may be decreased if not adjusted for energy. Wheat fiber reduces overall organic matter digestibility which contributes to reduced performance (Son et al., 2023). Also, their higher CP can replace intact protein sources and increased P concentration can lower the amount of added inorganic P to the diet. Wheat shorts and red dog may be included at higher levels without as much impact on performance as other wheat co-products. Overall, wheat co-products can be fed effectively in all stages of production.

### Co-Product Fiber and Gastrointestinal Health

The studies discussed in the current review focus on wheat co-product’s influence on gastrointestinal health with antibiotic-free diets. It is important to note that dietary antibiotics use will affect the response in pigs when used in combination or comparing among wheat co-products. Antibiotic use in swine diets is becoming less frequent because of the occurrence antibiotic resistance. This section provides an insight into how wheat co-products can influence gastrointestinal health in diets without antibiotics.

Historically, high-fiber ingredients were considered to have a lower nutritional value because of their lower energy or AA concentrations (Li et al., 2021). More recently, high-fiber ingredients are gaining attention because of their ability to lower diet cost and provide gastrointestinal benefits. Based on summarized mean nutrient values from the current review, wheat bran, middlings, and millrun are good sources of insoluble fiber containing 38.9%, 33.7%, and 35.5%, respectively. Although not as high as the other wheat co-products, wheat shorts and red dog still contain 15.2% and 12.8% insoluble fiber, respectively. In addition, wheat shorts also contain 4.1% soluble fiber.

Of all wheat co-products, wheat bran has the highest NDF, TDF, and insoluble fiber. Wheat bran has gained attention because of its potential benefits on gut health in weaned pigs (Koo et al., 2017). Wheat bran’s high insoluble fiber can act to inhibit pathogen adhesion by providing an alternative adhesion site (Molist et al., 2014). Molist et al. (2010) observed 4% wheat bran significantly reduced ileal *E. coli* concentration regardless of wheat bran particle size in nursery pigs challenged with *E. coli* K88^+^. The authors also observed pigs fed coarse wheat bran improved fecal consistency. In addition to wheat bran, wheat middlings are also a good source of insoluble fiber. Although not significant, Berrocoso et al. (2015) observed numeric benefits to feeding 2.5% and 5% wheat middlings in nursery pig diets compared to other fiber sources at the same inclusion rate under poor hygienic conditions. The authors observed wheat middlings had numerically improved villus height:crypt depth ratio and postweaning diarrhea presence compared to straw, oat hulls, and sugar beet pulp. Therefore, high insoluble fiber wheat co-products at 4% to 5% of the diet may be fed in nursery pig diets to improve gastrointestinal health parameters especially during enteric challenges.

High insoluble fiber in wheat co-products can also act to enhance proliferation of beneficial bacteria in the gastrointestinal tract of pigs (Heo et al., 2013). Wang et al. (2023b) observed nonpregnant sows fed 20% finely ground wheat bran were able to increase the abundance of Lactobacillaceae and concentrations of their metabolites such as short-chain fatty acids in the ileum. In addition, Leblois et al. (2017) observed 7% wheat bran fed to sows during gestation and lactation was able to modulate the piglet’s microbiota. Similar findings were reported by Li et al. (2023) where growing pigs fed 16% wheat bran had increased abundance of Streptococcus and Prevotellaceae bacteria which resulted in increased total antioxidant capacity of blood serum. Streptococcus bacteria contribute to short-chain fatty acid production, while Prevotellaceae maintain intestinal health and enhance immune and antioxidant function (Downes et al., 2013; Zhang et al., 2021). Liu et al. (2023) also observed improved serum antioxidant capacity and cecal short-chain fatty acid concentration in addition to improved gut morphology from pigs fed 17% wheat bran fiber. Little data is available on the effect other wheat co-products have on the microbiome. However, wheat middlings and millrun may lead to similar responses as wheat bran because of the high insoluble-to-soluble fiber ratio characteristics.

## Conclusion

Wheat and wheat co-products can offer certain benefits in swine diets such as high CP, AA, and P. In addition, wheat and wheat co-products are particularly appealing for pelleted diets because of improvements in pellet quality. When formulating with wheat and wheat co-products, it is important to know the class of wheat or the wheat co-product classification for accurate nutrient formulation. The current review provides a summarization of nutritional aspects associated with wheat and wheat co-products, where variation may occur, and their implementation into swine diets.
